# Supplementary material for: RALYL increases hepatocellular carcinoma stemness by sustaining the mRNA stability of TGF-β2
Source: Nat Commun. 2021 Mar 9;12:1518. doi: 10.1038/s41467-021-21828-7 (PMC7943813; doi:10.1038/s41467-021-21828-7)
Supplement: Supplementary file 1 — Supplementary Information [file 41467_2021_21828_MOESM1_ESM.pdf]

SUPPLEMENTARY INFORMATION

RALYL increases hepatocellular carcinoma stemness by sustaining the mRNA stability of TGF-β2

Xia Wang,[1, 2, 3] Jin Wang,[4] Yu-Man Tsui,[2,3] Chaoran Shi,[1, 2] Ying Wang,[1, 2, 5] Xin Zhang,[2,3] Qian Yan,[1, 2] Miao Chen,[1, 2] Chen Jiang,[1,6,7] Yun-Fei Yuan,[6] Chun-Ming Wong,[2,3] Ming Liu [1,2, 8] Zeng-yu Feng,[9] Honglin Chen,[10] Irene Oi Lin Ng, [2,3] Lingxi Jiang, [2, 9*] and Xin-Yuan Guan [1, 2*]

[1]Department of Clinical Oncology, [2]State key Laboratory of Liver Research, [3]Department of Pathology, and [4]School of Biomedical Sciences, Li Ka Shing Faculty of Medicine, The University of Hong Kong, Hong Kong, China. [5]Department of Radiation Oncology, [6]State Key Laboratory of Oncology in Southern China, and [7]Department of Pathology, Sun Yat-Sen University Cancer Center, Guangzhou, China. [8]Affiliated Cancer Hospital and Institute of Guangzhou Medical University, Guangzhou Municipal and Guangdong Provincial Key Laboratory of Protein Modification and Degradation, School of Basic Medical Sciences, Guangzhou Medical University, Guangzhou, China. [9]Department of General Surgery, Ruijin Hospital, Shanghai JiaoTong University School of Medicine, Shanghai, China. [10]Department of Microbiology, Li Ka Shing Faculty of Medicine, The University of Hong Kong, Hong Kong, China.

***Correspondence:** XY Guan, xyguan@hku.hk; LX Jiang, jlx12120@rjh.com.cn.

Supplementary Figures

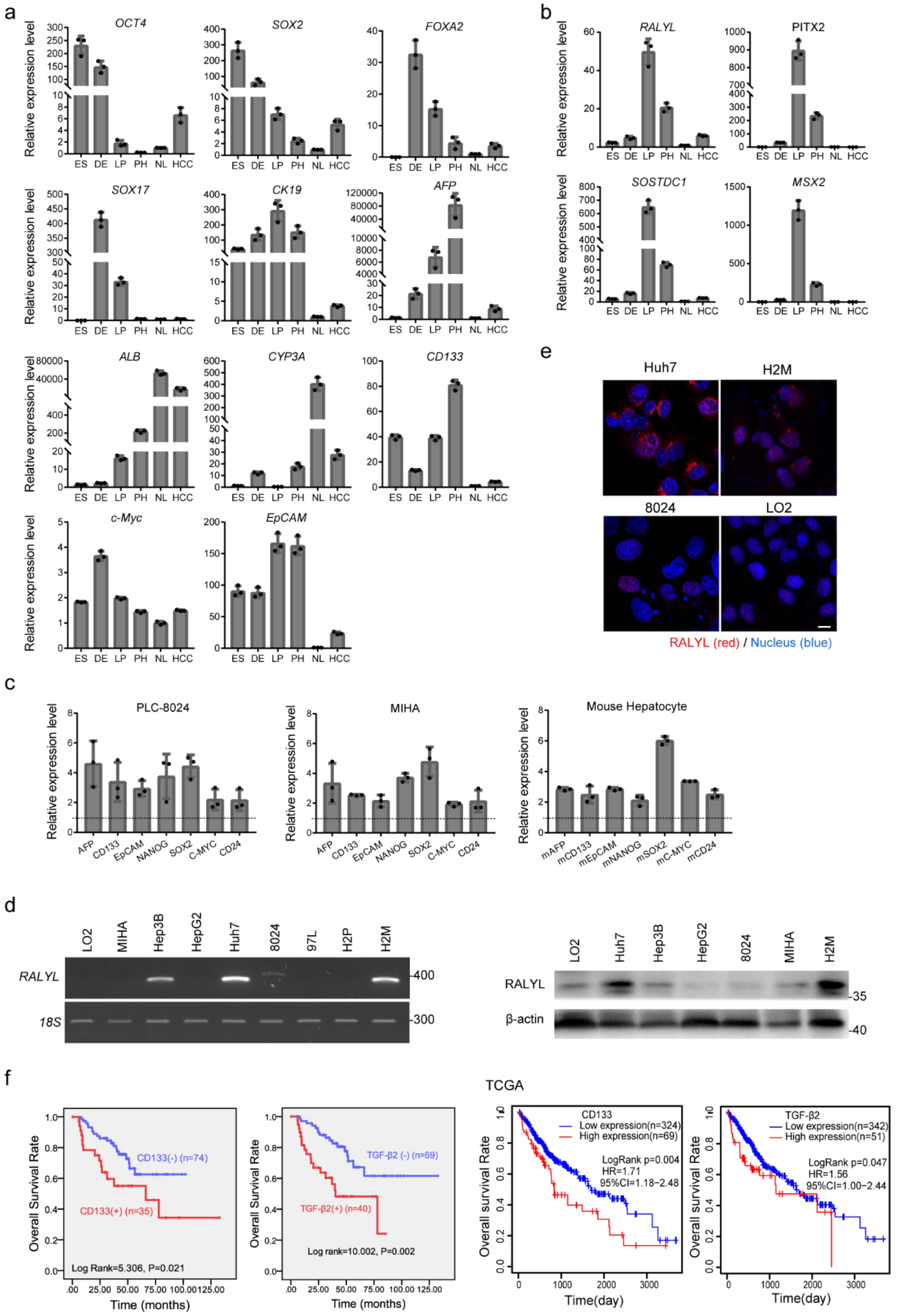

Wang et al. Supplementary Figure 1

Supplementary Figure 1. *RALYL* selection. (a) The expression pattern of *OCT4*, *SOX2*, *FOXA2*, *SOX17*, *CK19*, *AFP*, *ALB*, *CYP3A4*, *CD133*, *c-Myc* and *EpCAM* was validated using qRT-PCR to confirm the reliability of the hepatic differentiation model. The values represent the mean ± SD of 3 independent experiments (*P<0.05, **P<0.01, independent Student's *t* test). (b) The expression pattern of *RALYL* and other candidate genes, including *PITX2*, *SOSTDC1*, and *MSX2*, were confirmed using qRT-PCR. (c) qPT-PCR was performed to detect the relative expression levels of CSC- and stemness-related markers, including *AFP*, *CD133*, *NANOG*, *SOX2*, *c-Myc*, and *CD24*, in PLC-8024, MIHA and mouse hepatocyte with *RALYL* overexpression or not. The values represent the mean ± SD of 3 independent experiments (*P<0.05, **P<0.01, independent Student's *t* test). (d) Expression of *RALYL* in immortalized liver cell lines (MIHA and LO2) and HCC cell lines was determined using RT-PCR and western blotting. *18s* and β-actin were used as loading controls separately. (e) Immunofluorescence (IF) staining of *RALYL* (red) was performed in four cell lines: Huh7, H2M, PLC-8024, and LO2. DAPI (blue) was used for nuclei counterstaining. Scale bar = 10 μm. (f) Kaplan-Meier overall survival curve of two HCC groups in our in-house cohort or TCGA cohort: CD133(+) (red), patients with higher CD133 expression; CD133(–) (blue), patients with lower CD133 expression. TGF-β2(+) (red), patients with higher TGF-β2 expression; TGF-β2(–) (blue), patients with lower TGF-β2 expression.

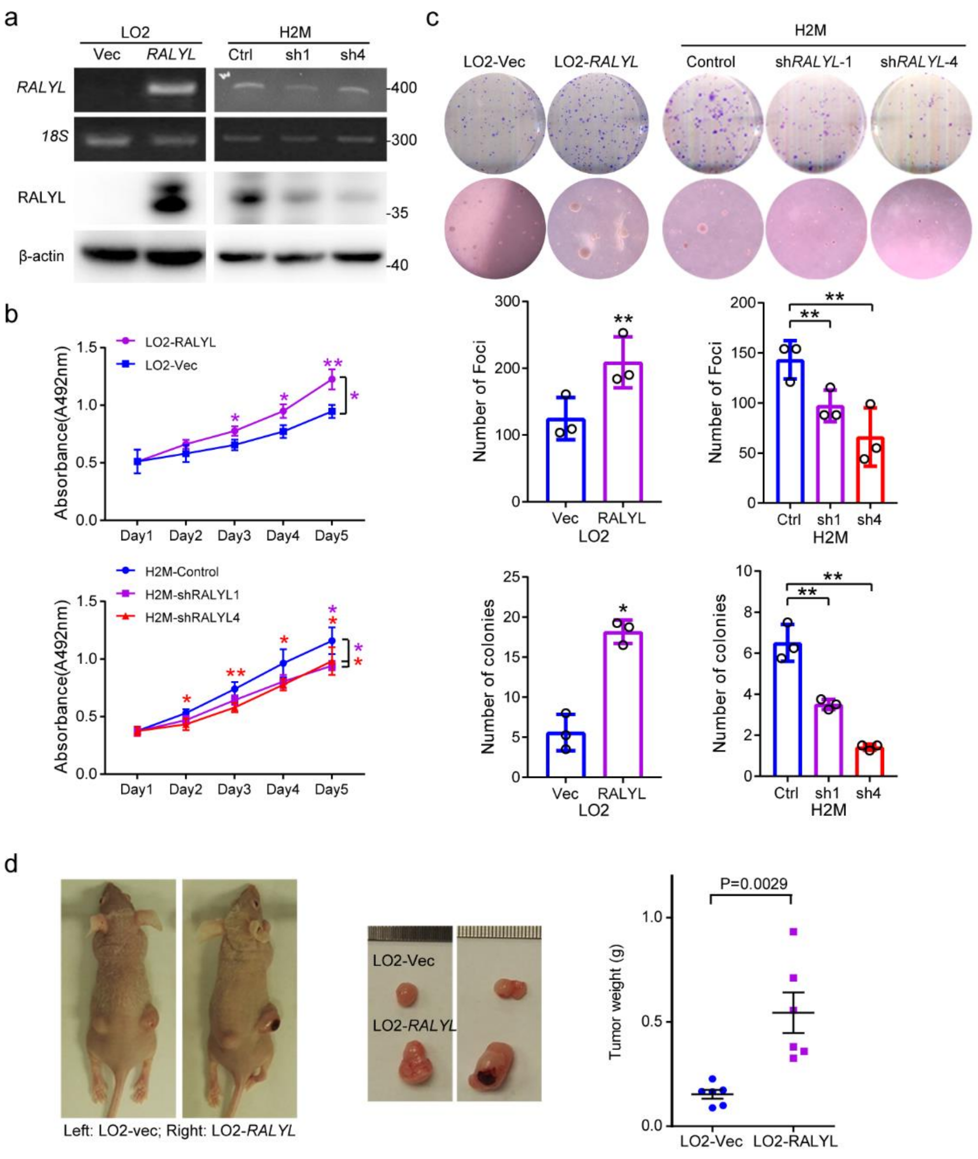

Wang et al. Supplementary Figure 2

Supplementary Figure 2. *RALYL* promotes the tumorigenic ability of HCC. (a) Ectopic expression of *RALYL* in *RALYL*- or Vec-transfected LO2 cells and *RALYL* silencing in H2M cells treated with scrambled shRNA (Ctrl: Control) or shRNA against *RALYL* (sh*RALYL*-1: sh1; sh*RALYL*-4: sh4) were confirmed using RT-PCR and western blotting. *18S* and β-actin were used as loading controls separately. (b) The cell growth rates were examined with the XTT assay. The values represent the mean ± SD of 3 independent experiments. *P* value was shown as *(P< 0.05) or **(P < 0.01) at some time points and highlighted in the color same with the line of corresponding experiment

group (purple asterisks represent LO2-*RALYL* *vs* LO2-Vec, H2M-sh*RALYL*1 *vs* H2M-Control; red asterisks represent H2M-sh*RALYL*4 *vs* H2M-Control). (c) Representative images of foci formation (up) and colony formation in soft agar (low) induced by LO2-Vec/*RALYL* and H2M-Control/sh*RALYLs*. The numbers of foci and colonies are shown in the bar chart below. Values indicate the mean standard deviation of 3 independent experiments (**P* <0.05, ***P* < 0.01, two-sided Student's t-test). (d) Representative images of mice with tumors induced by LO2-Vev/*RALYL*. Tumor weights are summarized in the dot plot (right) which express as mean ± SD of 6 mice.

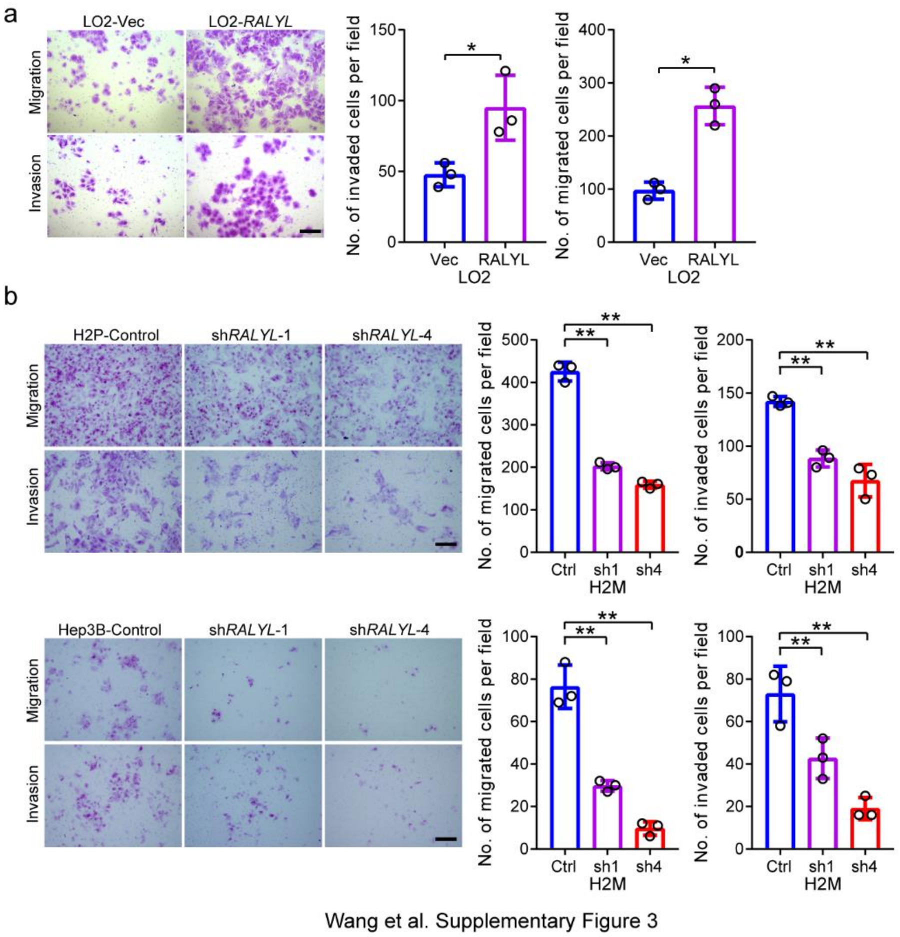

Supplementary Figure 3. *RALYL* promotes HCC migration and invasion. Cell motilities of LO2-Vec/*RALYL* (a), H2M-Control/sh*RALYLs*, and Hep3B-Control/sh*RALYLs* (b) were assessed using transwell migration and matrigel invasion assays. The number of migrated or invaded cells is summarized in the bar chart. Data are presented as mean ± SD of 3 independent experiments (*P <0.05, **P <0.01, two-sided Student's t-test). Scale bar = 200 μm.

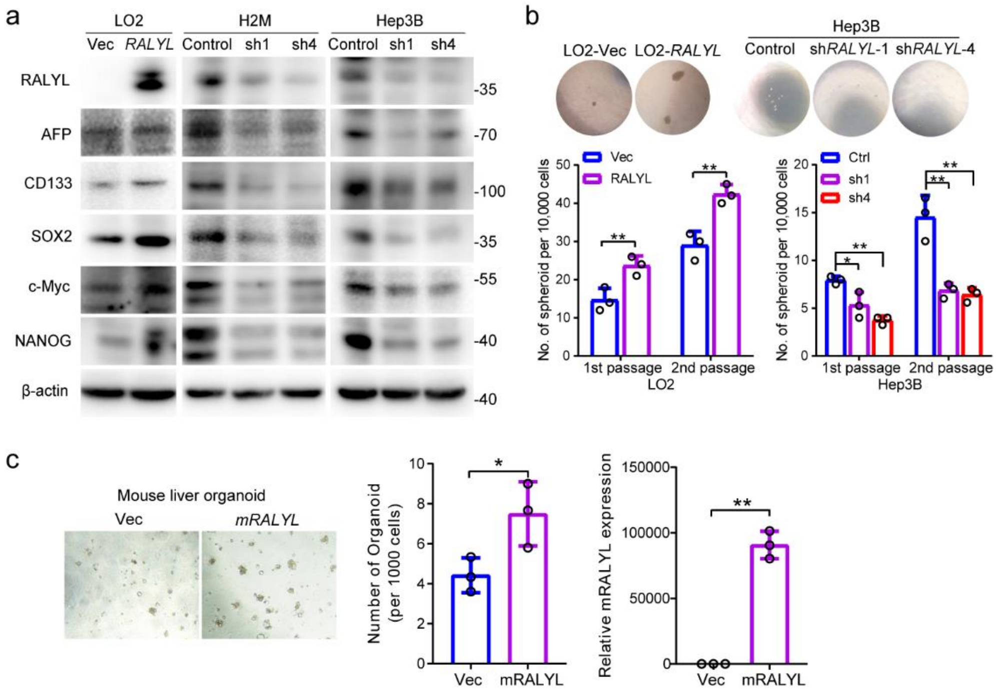

Wang et al. Supplementary Figure 4

Supplementary Figure 4. ***RALYL*** **enhances stemness features of HCC cells.** (a) The expression of stemness markers in indicated *RALYL*-overexpression cells and *RALYL*-silencing cells were determined by western blotting. β-actin was used as the loading control. (b) The self-renewal ability of LO2-Vec/*RALYL* and Hep3B-Control/sh*RALYLs* was assessed using the spheroid formation assay. The numbers of primary and secondary spheroids were summarized in the bar chart. Values represent the mean ± SD of 3 independent experiments. (***P*<0.01, two-sided Student's t-test). (c) Mouse liver organoid was overexpressed with *mRALYL*. The numbers of organoid were calculated and shown in the bar chart. The expression level of *mRALYL* was validated by qRT-PCR. Data are presented as mean ± SD of 3 independent experiments (*P<0.05, **P<0.01, two-sided Student's t-test).

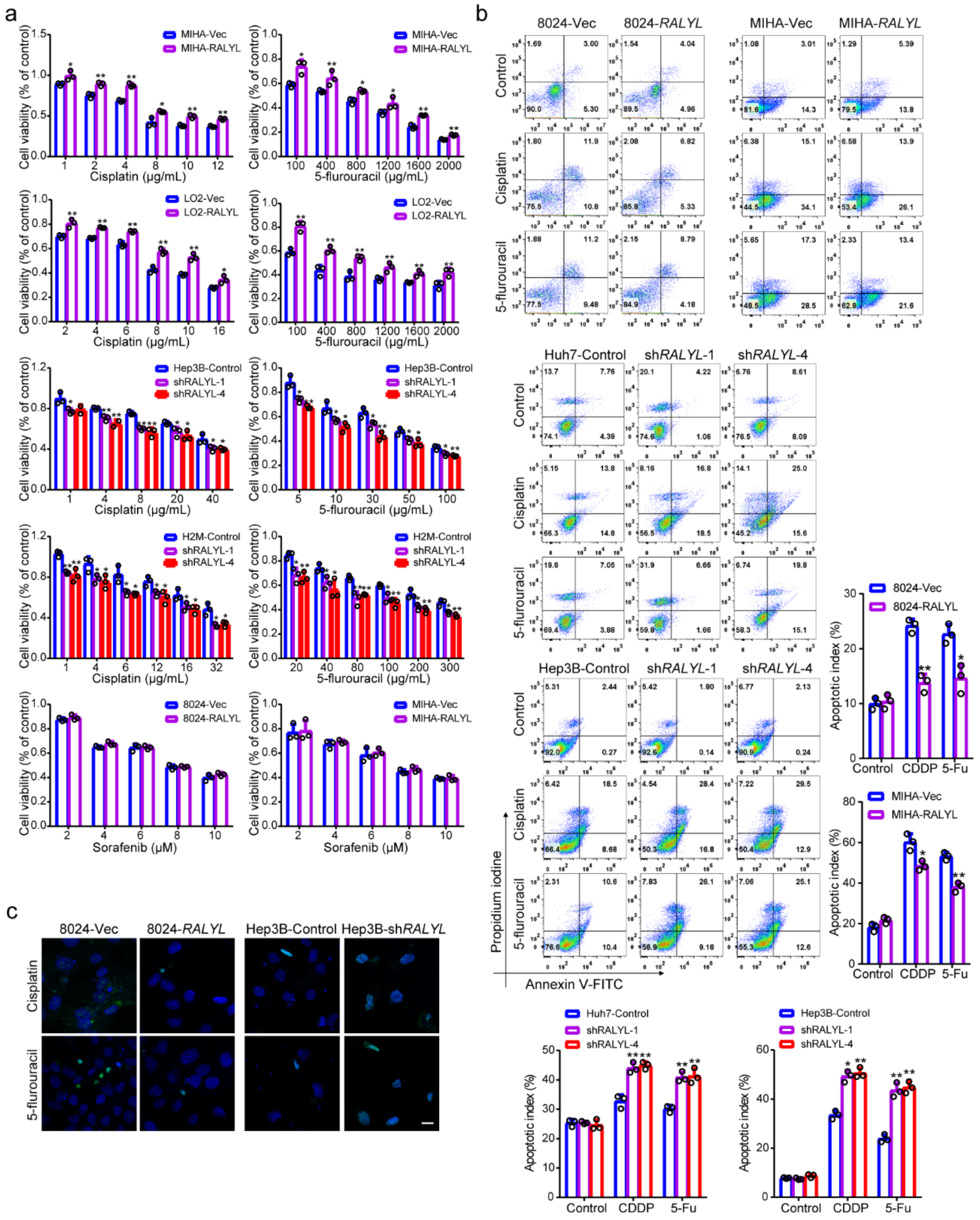

Wang et al. Supplementary Figure 5

Supplementary Figure 5. *RALYL* enhances chemoresistant ability of HCC cells. (a) The XTT assay was performed to assess the chemoresistance of MIHA-Vec/*RALYL*, LO2-Vec/*RALYL*, H2M-Control/sh*RALYL*, and Hep3B-Control/sh*RALYL* cells to the indicated concentration of CDDP or 5-Fu for 48 h. The results are presented as mean ± SD of 3 independent tests (**P*<0.05, ***P*<0.01). (b) Representative images of apoptosis

analysis of CDDP or 5-Fu treated 8024-Vec/*RALYL*, MIHA-Vec/*RALYL*, Huh7-Control/sh*RALYL*, and Hep3B-Control/sh*RALYL*. The apoptotic index was shown in the bar chat with values represented as mean ± SD of 3 independent tests (Right) (*P<0.05; **P<0.01). (c) Representative images of TUNEL assay which is performed in CDDP and 5-Fu treated 8024-Vec/*RALYL* and Hep3B-Control/sh*RALYL* cells. Scale bar = 20μm.

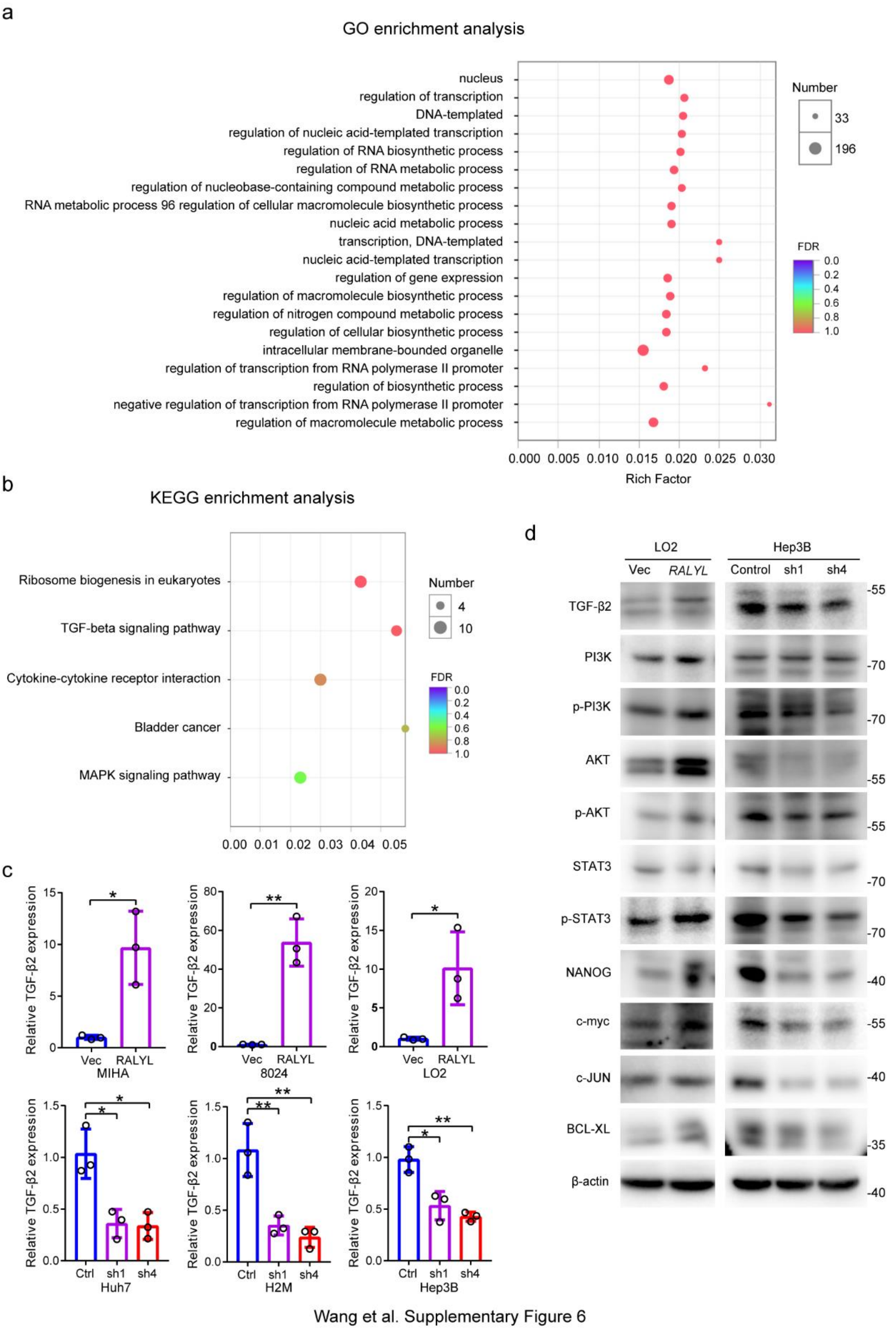

Supplementary Figure 6. ***RALYL*** **enhances the stemness of HCC through TGF-β2 signaling.** Transcriptome sequencing analysis was applied to compare different gene

expression between 8024-*RALYL* and 8024-Vec. Gene Ontology (GO) enrichment analysis (a) and KEGG enrichment analysis (b) were used to identify the functions of *RALYL* by analyzing genes upregulated by *RALYL*. Colors from blue to red represent combined enrichment score from low to high. Size indicates number of significant regulated genes per total genes in GO-Term or KEGG-Term. (c) The expression level of TGF-β2 in 8024-Vec/*RALYL*, MIHA-Vec/*RALYL*, LO2-Vec/*RALYL*, Huh7-Control/sh*RALYLs*, H2M-Control/sh*RALYLs*, and Hep3B-Control/sh*RALYLs* was determined by qRT-PCR. The values represent the mean ± SD of 3 independent experiments. (*$P<0.05$, **$P<0.01$, independent Student's t test). (d) Western blotting was performed to compare the levels of PI3K, p-PI3K, AKT, p-AKT, STAT3, p^{Y705}STAT3, NANOG, c-Myc, c-Jun, and BCL-XL in cell lysates from LO2-Vec/*RALYL* and Hep3B-Control/sh*RALYLs*. β-actin was used as the loading control.

Supplementary Table 1. Association between *CD133* expression and clinicopathologic features in 117 HCC cases.

Features	Total	*CD133* expression*		*P*-value
		Low	High	
Sex				0.859
Male	98	65	33	
Female	19	13	6	
Age, years				0.609
≤60	96	61	35	
>60	21	17	4	
Serum AFP, ng/mL				0.148
≤400	65	47	18	
>400	52	31	21	
Serum HBsAg				0.849
Negative	16	11	5	
Positive	101	67	34	
Cirrhosis				0.735
Absent	36	25	11	
Present	80	53	27	
Differentiation				**0.034**
Well/moderate	67	50	17	
Poor	50	28	22	
Tumor size				0.085
≤5	49	37	12	
>5	68	41	27	
TNM stage (AJCC)				1.000
I	84	56	28	
II/III	33	22	11	
Vascular invasion				0.286
Absent	102	70	32	
Present	13	7	6	
Metastasis				**0.050**
Absent	57	43	14	
Present	60	35	25	

Statistical significance ($P \leqslant 0.05$) is shown in bold.

Pearson $\chi 2$ test.

#Partial data are not available, and the statistic was based on available.

Supplementary Table 2. Association between *TGF-β2* expression and clinicopathologic features in 117 HCC cases.

Features		Total	*TGF-β2* expression*		*P*-value
			Low	High	
Sex					0.721
	Male	98	61	37	
	Female	19	11	8	
Age, years					0.648
	≤60	96	60	36	
	>60	21	12	9	
Serum AFP, ng/mL					0.702
	≤400	65	39	26	
	>400	52	33	19	
Serum HBsAg					0.932
	Negative	16	10	6	
	Positive	101	62	39	
Cirrhosis					0.272
	Absent	36	25	11	
	Present	80	47	33	
Differentiation					**0.009**
	Well/moderate	67	48	19	
	Poor	50	24	26	
Tumor size					0.477
	≤5	49	32	17	
	>5	68	40	28	
TNM stage (AJCC)					0.162
	I	84	55	29	
	II/III	33	17	16	
Vascular invasion					**0.014**
	Absent	102	67	35	
	Present	13	4	9	
Metastasis					**0.024**
	Absent	57	41	16	
	Present	60	31	29	

Statistical significance ($P \leqslant 0.05$) is shown in bold.

Pearson $\chi 2$ test.

#Partial data are not available, and the statistic was based on available.

Supplementary Table 3. List of primers used in this study.

Gene	Primer	Sequence
For Real-time PCR		
RALYL	Forward	GCGCCTGGAGAAGATTGAGA
	Reverse	GAAACAGCTCATGACCCCCA
CD133	Forward	GTGCGGGAACTCCTTTTCAG
	Reverse	TCTTCTGGGAAATCACGCGG
TGF-β2	Forward	GTTACAACACCCTCTGGCTCA
	Reverse	TGTTCAGGCACTCTGGCTTT
CK8	Forward	AGGACCTGCAGGAAGGGATCT
	Reverse	GCCGCCTAAGGTTGTTGATG
CK18	Forward	GAGGGCTCAGATCTTCGCA
	Reverse	CCAGCTGCAGTCGTGTGATA
Albumin	Forward	GGTTGATGTGATGTGCACTG
	Reverse	AGTCTCTGTTTGGCAGACGA
AFP	Forward	ACAAAAAGCCCACTCCAGCA
	Reverse	ATGGCTTGGAAAGTTCGGGT
NANOG	Forward	AATACCTCAGCCTCCAGCAGATG
	Reverse	TGCGTCACACCATTGCTATTCTTC
GAPDH	Forward	GGAGCGAGATCCCTCCAAAAT
	Reverse	GGCTGTTGTCATACTTCTCATGG

Supplementary Table 4. List of antibodies used in this study.

Antibody	Size (kDa)	Vendor[a]	Cat No.	Application
Rabbit anti-human RALYL	36	Sigma	HPA055868	WB, 1:1000; IF, 1:1000; IHC: 1:500
Mouse anti-human β-actin	43	Abcam	ab6276	WB, 1:5000
Rabbit anti-human CD133	110	Abcam	ab19898	WB, 1;1000
Mouse anti-human AFP	70	Abcam	ab3980	WB, 1;1000
Rabbit anti-human NANOG	42	Cell signaling	#4903	WB, 1;1000
Rabbit anti-human SOX2	34	Abcam	ab92494	WB, 1;1000
Mouse anti-human c-Myc	50	Invitrogen	132500	WB, 1;1000
Rabbit anti-human TGF-β2	50	Abcam	ab36495	WB, 1;1000
Mouse anti-human STAT3	88	Cell signaling	#9139	WB, 1;1000
Rabbit anti-human phospho-STAT3 (Tyr705)	88	Abcam	ab76315	WB, 1;1000
Rabbit anti-human Akt	60	Cell signaling	#4691	WB, 1;1000
Rabbit anti-human phospho-Akt (Ser473)	60	Cell signaling	#9271	WB, 1;1000
Mouse anti-human E-cadherin	135	Cell signaling	#3195	WB, 1;1000
Mouse anti-human Fibronectin	285	Abcam	ab2413	WB, 1;1000
Rabbit anti-human Bcl-XL	30	Cell signaling	#2762	WB, 1;1000
Rabbit anti-human snail	29	Cell signaling	#3879	WB, 1;1000
Rabbit anti-human slug	30	Cell signaling	#9585	WB, 1;1000
Rabbit anti-human FTO	60	Cell signaling	#31687	WB, 1;1000
Mouse anti-human CD133-PE	110	Miltenyi Biotec	130-090-853	Flow cytometry, 1:100
Rabbit anti-N6-methyladenosine		Synaptic System	#202003	RIP: 15μg

[a]Cell Signaling Technology, Danvers, MA, USA

Invitrogen, Carlsbad, California

Abcam, Cambridge, MA, USA

Santa Cruze, CA, USA

Sigma-Aldrich, St. Louis, MO, USA

Supplementary Methods

Tumorigenicity assays

For the cell proliferation assay, 1,000 cells were seeded in each well of 96-well plates, and the cell growth rate was assessed using the XTT kit (Roche Diagnostics, Indianapolis, IN). The foci formation assay was used to assess anchorage-dependent growth. In brief, 1,000-2,000 cells were seeded in each well of six-well plates for approximately 2-3 weeks. Surviving colonies were stained and counted using crystal violet (Sigma-Aldrich, St. Louis, MO). Anchorage-independent growth was assessed by colony formation in soft agar. Approximately 1,000-3,000 cells were seeded in 0.35% bacto-agar on a bottom layer of solidified 0.5% bacto-agar in six-well plates. After culturing for 3-4 weeks, colonies more than 50 cells were counted and described as the mean ± SD of three independent experiments.

The *in vivo* mouse xenograft model was used to assess *in vivo* tumorigenicity using subcutaneous injection. In brief, 3×10^6 PLC-8024 cells or 5×10^6 MIHA, LO2, Huh7, and Hep3B cells were subcutaneously injected into the left or right side of the dorsal flank of 4-5-week-old BALB/cAnN-nu (nude) mice. After 4-6 weeks, tumor formation was assessed, and the mice were killed. All animal experiments were conducted and approved by the University of Hong Kong Committee on the Use of Live Animals in Teaching and Research.

Cell motility assays

Transwell migration and invasion assays were performed to evaluate cell motility (BD Biosciences, San Jose, CA). Approximately 5×10^4 cells were seeded in the medium without FBS on transwell upper chambers, and the lower chamber was supplied with medium with 10% FBS. The cells that migrated and invaded to the lower membrane surface were stained using crystal violet and then counted under a microscope.

***In vivo* liver metastasis models**

Nude mice were intrasplenically injected with HCC cells to assess the liver metastatic capacity *in vivo*. After surgery, mice recovered from anesthesia in a cage under a heater. Analgesia was provided to the nude mice during the first postoperative week. Twelve weeks later, all mice were killed, and the livers, spleens, and lungs were excised and fixed in 4% paraformaldehyde overnight. Thereafter, fixed tissues were embedded in paraffin for further studies.

Spheroid formation assay

A total of 1,000 cells were cultured in the DMEM/F12 medium (Life Technologies,) supplemented with 20ng/mL EGF (Life Technologies), 10ng/mL basic FGF (Life Technologies), B27 (1:50, GIBCO), and 4μg/mL insulin (BIOIND, Kibbutz Beit Haemek, Israel) in 24-well plates, which are coated with poly HEMA (Sigma-Aldrich). The cells were replenished with 30-μL supplementary medium every other day.

Differentiation assay

The cells were treated with all-trans retinoic acid (atRA) (Sigma-Aldrich) for 5 days at 20 or 30 mM. The medium was changed every day. Expression changes of mature

hepatocyte markers, CK18, CK8, and albumin, and stemness-related markers, AFP, NANOG, and CD133, as well as *RALYL*, were assessed using RT-PCR.
