# Supplementary material for: RALYL increases hepatocellular carcinoma stemness by sustaining the mRNA stability of TGF-β2
Source: Nat Commun. 2021 Mar 9;12:1518. doi: 10.1038/s41467-021-21828-7 (PMC7943813; doi:10.1038/s41467-021-21828-7)
Supplement: Supplementary file 3 — Reporting Summary [file 41467_2021_21828_MOESM3_ESM.pdf]

nature research

Corresponding author(s): Xin-Yuan GUAN

Last updated by author(s): 05/02/2021

Reporting Summary

Nature Research wishes to improve the reproducibility of the work that we publish. This form provides structure for consistency and transparency in reporting. For further information on Nature Research policies, see our Editorial Policies and the Editorial Policy Checklist.

Statistics

For all statistical analyses, confirm that the following items are present in the figure legend, table legend, main text, or Methods section.

n/a	Confirmed	
☐	☒	The exact sample size (*n*) for each experimental group/condition, given as a discrete number and unit of measurement
☐	☒	A statement on whether measurements were taken from distinct samples or whether the same sample was measured repeatedly
☐	☒	The statistical test(s) used AND whether they are one- or two-sided<br>*Only common tests should be described solely by name; describe more complex techniques in the Methods section.*
☐	☒	A description of all covariates tested
☐	☒	A description of any assumptions or corrections, such as tests of normality and adjustment for multiple comparisons
☐	☒	A full description of the statistical parameters including central tendency (e.g. means) or other basic estimates (e.g. regression coefficient) AND variation (e.g. standard deviation) or associated estimates of uncertainty (e.g. confidence intervals)
☐	☒	For null hypothesis testing, the test statistic (e.g. *F*, *t*, *r*) with confidence intervals, effect sizes, degrees of freedom and *P* value noted<br>*Give P values as exact values whenever suitable.*
☒	☐	For Bayesian analysis, information on the choice of priors and Markov chain Monte Carlo settings
☐	☒	For hierarchical and complex designs, identification of the appropriate level for tests and full reporting of outcomes
☐	☒	Estimates of effect sizes (e.g. Cohen's *d*, Pearson's *r*), indicating how they were calculated

Our web collection on statistics for biologists contains articles on many of the points above.

Software and code

Policy information about availability of computer code

Data collection	No software was used for data collection.
Data analysis	X-Tile software (V3.6.1) was used for determine the optimal cut-of value of HCC samples from TCGA database. Kaplan–Meier (K-M) survival curves were plotted by the 'survival' package (version 2.44-1.1) in the R environment (version 3.5.2). R code is available upon request.<br>FlowJo_V10 for analysis of flow cytometry data.<br>SPSS _V19 for clinical significances and survival analysis of our in-house cohort.<br>ELDA software is a webtool which is available at [http://bioinf.wehi.edu.au/software/elda/].<br>Statistical analysis was performed by Prism 7 Graphpad Software.

For manuscripts utilizing custom algorithms or software that are central to the research but not yet described in published literature, software must be made available to editors and reviewers. We strongly encourage code deposition in a community repository (e.g. GitHub). See the Nature Research guidelines for submitting code & software for further information.

Data

Policy information about availability of data

All manuscripts must include a data availability statement. This statement should provide the following information, where applicable:

- Accession codes, unique identifiers, or web links for publicly available datasets
- A list of figures that have associated raw data
- A description of any restrictions on data availability

All data supporting the findings of this study are available within the paper and its supplementary information files. The raw and processed sequencing data are available in Gene Expression Omnibus under accession GSE163601. The source data underlying Figs. 1d, 2b–e, 3a-c, 4b and d-f, 5e and f, 6a–d and Supplementary Figs 1a-c, 2b–d, 3a and b, 4b-d, and 5c are provided as a Source Data file. All relevant data are available from the authors on reasonable request.

Field-specific reporting

Please select the one below that is the best fit for your research. If you are not sure, read the appropriate sections before making your selection.

- [x] Life sciences
- [ ] Behavioural & social sciences
- [ ] Ecological, evolutionary & environmental sciences

For a reference copy of the document with all sections, see nature.com/documents/nr-reporting-summary-flat.pdf

Life sciences study design

All studies must disclose on these points even when the disclosure is negative.

Sample size	The sample size was determined based on availability of patient samples. No sample size calculation was performed, but the current number of samples in the study was sufficient to derive statistical analysis. Overall, 117 pairs of primary HCC and their adjacent normal specimens were used for clinical analysis.<br>At least three repeats were performed in vivo and vitro functional assays for statistical analyses.
Data exclusions	No data was excluded from the analyses.
Replication	All the experiments were replicated. Generally, three independent experiments were carried out and each experiment was performed with three repeats.
Randomization	BALB/cAnN-nu (nude) mice were allocated into RALYL overexpression or knockdown group and control group randomly. Randomization was not possible for in vitro assays involving HCC cell lines
Blinding	Investigators were blinded to RALYL expression and no RALYL expression group during clinical data collection and clinicopathological association analysis.<br>For in vivo and in vitro functional assays., the investigators were not blinded to group allocation during experiments as data collection, analysis and outcome assessment required that the investigators were not blinded.

Reporting for specific materials, systems and methods

We require information from authors about some types of materials, experimental systems and methods used in many studies. Here, indicate whether each material, system or method listed is relevant to your study. If you are not sure if a list item applies to your research, read the appropriate section before selecting a response.

Materials & experimental systems

n/a	Involved in the study	
[ ]	[x]	Antibodies
[ ]	[x]	Eukaryotic cell lines
[x]	[ ]	Palaeontology and archaeology
[ ]	[x]	Animals and other organisms
[ ]	[x]	Human research participants
[x]	[ ]	Clinical data
[x]	[ ]	Dual use research of concern

Methods

n/a	Involved in the study	
[x]	[ ]	ChIP-seq
[ ]	[x]	Flow cytometry
[x]	[ ]	MRI-based neuroimaging

Antibodies

Antibodies used	The antibodies used are listed in Supplementary Table 4. The detail information of antibodies, such as Supplier name, catalog number is also shown in supplement table 4
Validation	All antibodies were obtained from commercial sources, with extensive validation for use in human specimens for each application by the vendor, and also by other researchers. Antibodies have also been tested extensively in other studies within the lab as well as several pilot studies.

Eukaryotic cell lines

Policy information about cell lines

Cell line source(s)	Human immortalized liver cell lines, i.e., MIHA and LO2, and HCC cell lines, i.e., Huh7, Hep3B, H2M, PLC-8024 were used in this study. HCC cell lines Huh7, Hep3B, and PLC-8024 were bought from ATCC, and the rest of the cell lines were bought from the cell bank of the Chinese Academy of Sciences.
Authentication	All cell lines used in this study were authenticated by morphological observation (MycoAlert; Lonza, Rockland,

ME).Furthermore, cell sorting by flow cytometry was performed on HCC cells using PE-conjugated monoclonal mouse anti-human CD133 and EPCAM to check CD133 and EPCAM expression. Their expression is consistent with previous reports in those cell lines.

Mycoplasma contamination	All cell lines used in this study have been tested for absence of mycoplasma contamination.
Commonly misidentified lines (See ICLAC register)	None misidentified lines has been used in this study.

Animals and other organisms

Policy information about studies involving animals; ARRIVE guidelines recommended for reporting animal research

Laboratory animals	4-5-week-old male BALB/cAnN-nu (nude) mice were used in this study. After 4-10 weeks, tumor formation was assessed, and the mice were killed and the tumors were dissected. The BALB/cAnN-nu (Nude) mice were maintained in a specific pathogen-free animal facility at the University of Hong Kong under 12-h light dark cycles, controlled temperature (~22°C), and 40–60% humidity with free access to food and water.
Wild animals	The study did not involve wild animals.
Field-collected samples	The study did not involve samples collected from the field.
Ethics oversight	All animal experiments were conducted and approved by the University of Hong Kong Committee on the Use of Live Animals in Teaching and Research (CULATR).

Note that full information on the approval of the study protocol must also be provided in the manuscript.

Human research participants

Policy information about studies involving human research participants

Population characteristics	117 pairs of primary HCC and their adjacent normal specimens were obtained from patients after hepatectomy at Sun Yat-Sen University Cancer Center (Guangzhou, China).The age distribution of HCC patients enrolled in the study is as follows: ≤60 years old (82%, 96/117), >60 years old (18%, 21/117). Male patients enrolled in the study constitute 84% of the total patient population (98/117) and female patients constitute 16% of the total population (19/117). 56% (65/117) of the HCC patients were diagnosed with ≤400ng/mL AFP and 44% were diagnosed with >400 ng/mL AFP in serum. 14% (16/117) were serum HBsAg negative and 86% (101/117) were serum HBsAg positive. 31% (36/117) were serum absent of cirrhosis and 69% (80/117) were present with cirrhosis. 57% (67/117) were well/moderate differentiation and 43% (50/117) were poor differentiation. 72% (84/117) were diagnosed with TNM satge [illegible] and 28% (33/117) were diagnosed with TNM satge [illegible] /[illegible] (AJCC). 89% (102/115) were absent of vascular invasion and 11% (13/115) were present of vascular invasion (two patients' information about vascular invasion were censored). 49% (57/117) were absent of metastasis and 61% (60/117) were present of metastasis.
Recruitment	Paitents who were diagnosed with HCC and performed hepatectomy at Sun Yat-Sen University Cancer Center with written informed consent in this study. The surgeons and pathologists were blinded in the study during data collection and examination. No self-selection bias was found present in the study.
Ethics oversight	Clinical specimens used in this study were approved by the Committee for Ethical Review of Research Involving Human objects at the Sun Yat-Sen University Cancer Center.

Note that full information on the approval of the study protocol must also be provided in the manuscript.

Flow Cytometry

Plots

Confirm that:

- [x] The axis labels state the marker and fluorochrome used (e.g. CD4-FITC).
- [x] The axis scales are clearly visible. Include numbers along axes only for bottom left plot of group (a 'group' is an analysis of identical markers).
- [x] All plots are contour plots with outliers or pseudocolor plots.
- [x] A numerical value for number of cells or percentage (with statistics) is provided.

Methodology

Sample preparation	After treating with CDDP or 5-FU for 48 h, the cells were collected and double stained with FITC-conjugated Annexin-V and PI provided in the BD apoptosis detection kit (BD Biosciences), thereby used for flow cytometry.
Instrument	Samples were sorted using the FACS Aria I Cell Sorter (BD Biosciences); The apoptotic assays were performed using the FACSCanto II Analyzer (BD Biosciences).
Software	FlowJo software (Tree Star) was used to analyze the flow cytometry data.

Cell population abundance	The PE-conjugated isotype mouse immunoglobulin G1b (MiltenyiBiotec) was used as the control.
Gating strategy	For positively stained cells, only the top 15% most brightly stained were selected as CD133-positive populations. Meanwhile, the bottom 15% most dimly stained cells were selected as CD133-negative cells.

☒ Tick this box to confirm that a figure exemplifying the gating strategy is provided in the Supplementary Information.
