# Supplementary material for: RALYL increases hepatocellular carcinoma stemness by sustaining the mRNA stability of TGF-β2
Source: Nat Commun. 2021 Mar 9;12:1518. doi: 10.1038/s41467-021-21828-7 (PMC7943813; doi:10.1038/s41467-021-21828-7)
Supplement: Supplementary file 4 — Source data file [file 41467_2021_21828_MOESM4_ESM.zip › Wang et al. Source Data/Wang et al. uncropped versions of gels or blots.pptx]

## Slide 1
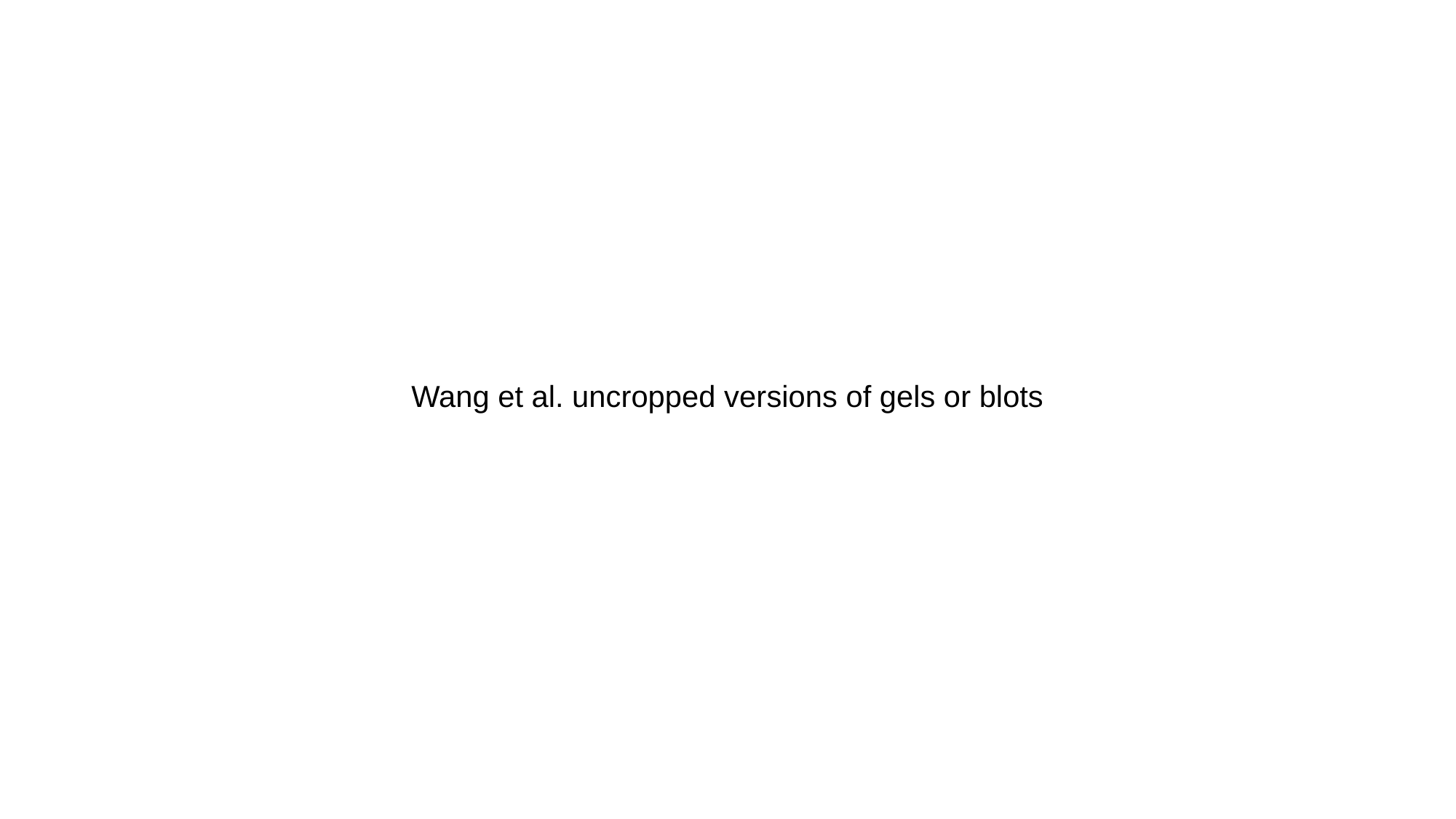

# Wang et al. uncropped versions of gels or blots

## Slide 2
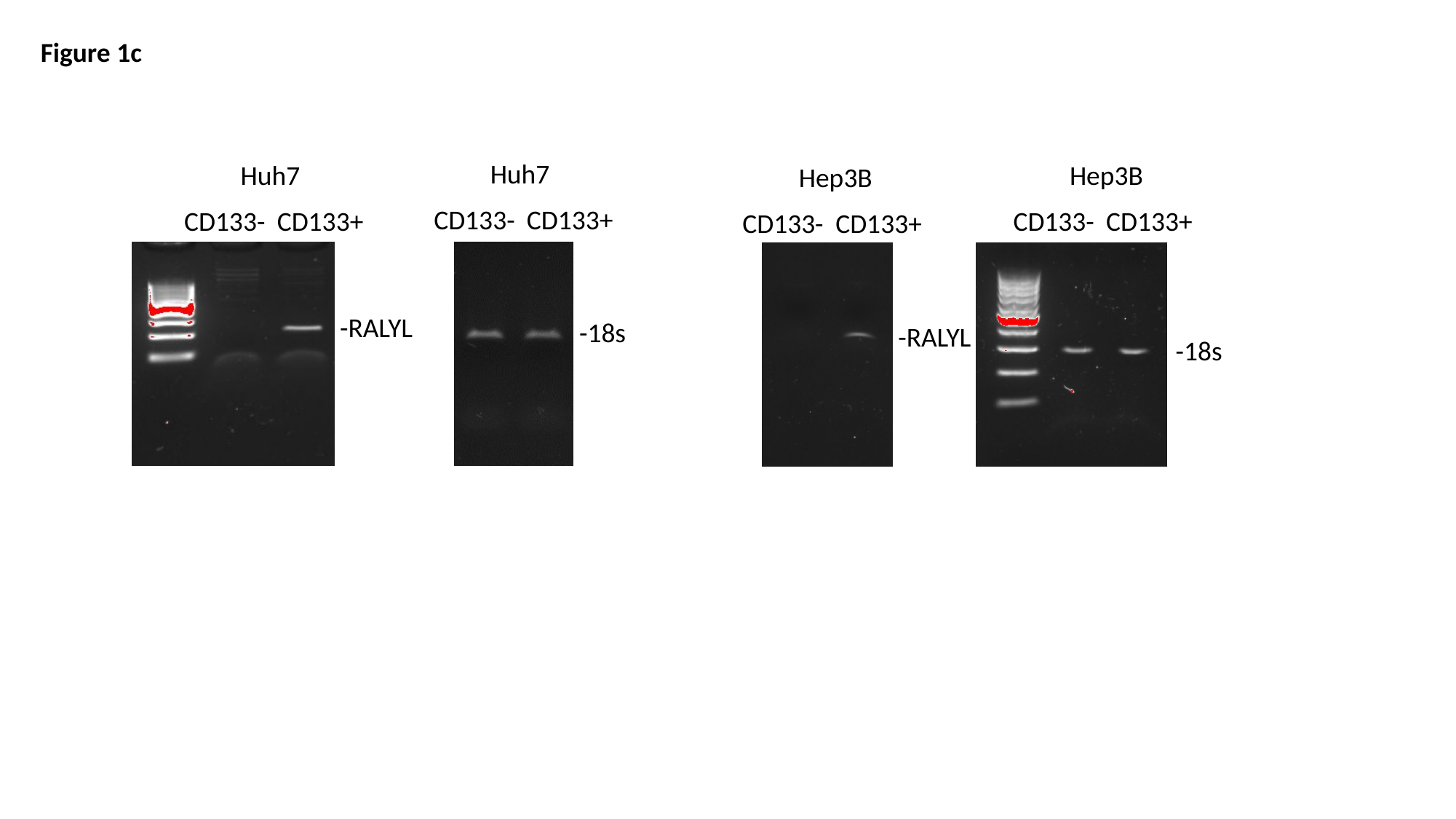

Figure 1c
Huh7
Huh7
Hep3B
Hep3B
CD133-
CD133+
CD133-
CD133-
CD133+
CD133+
CD133-
CD133+
-RALYL
-18s
-RALYL
-18s

## Slide 3
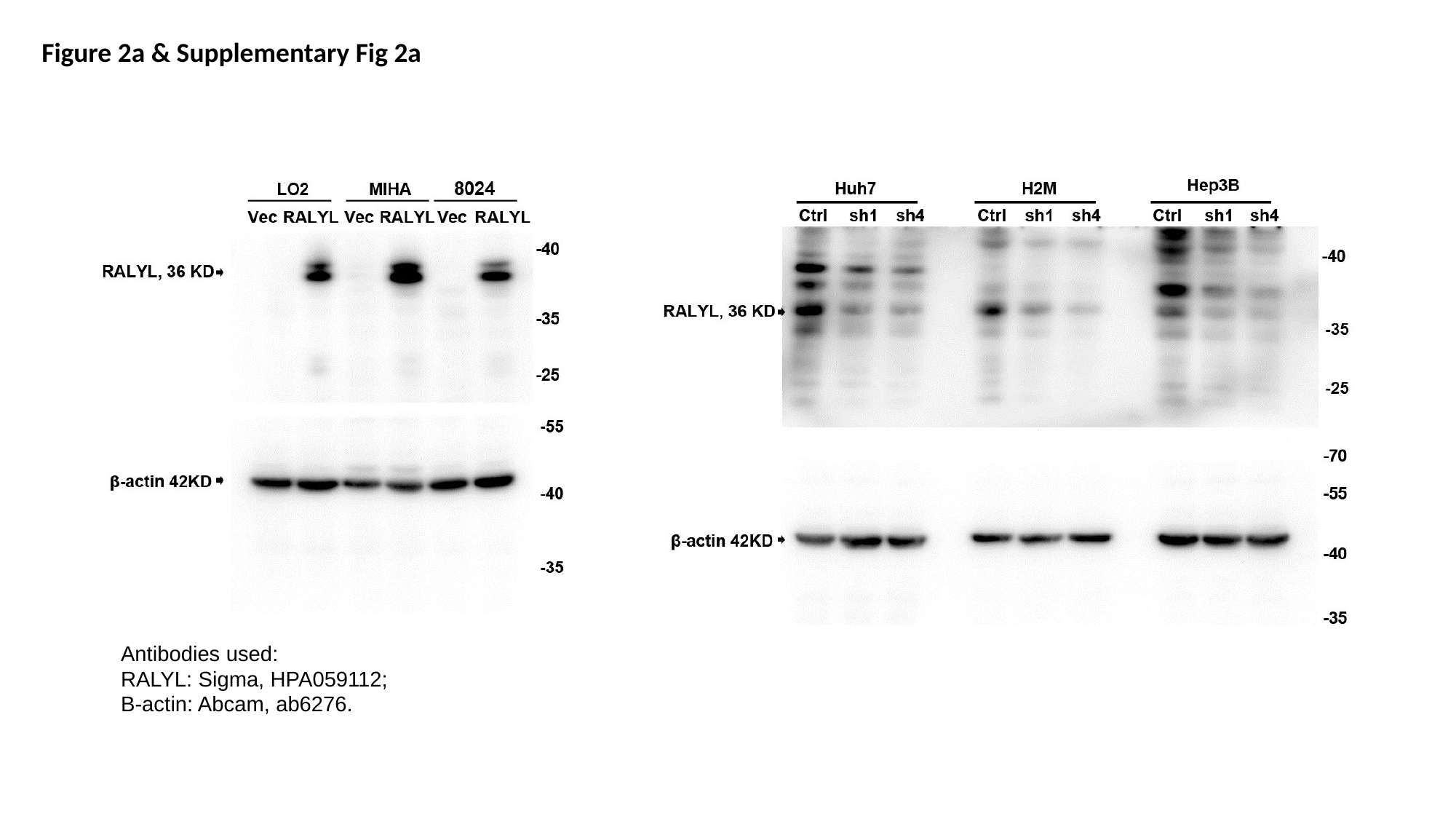

Figure 2a & Supplementary Fig 2a
Antibodies used:
RALYL: Sigma, HPA059112;
Β-actin: Abcam, ab6276.

## Slide 4
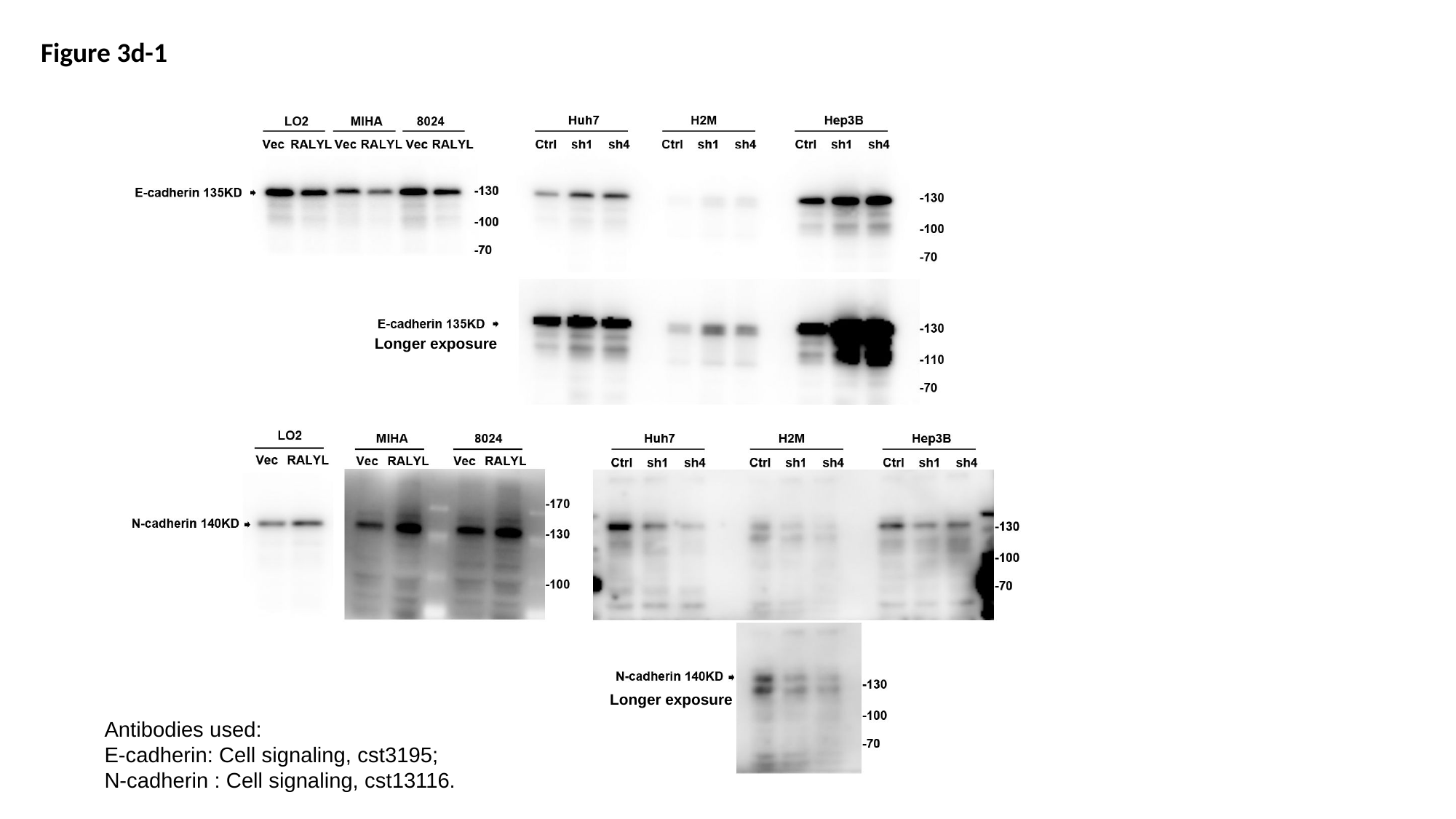

Figure 3d-1
Longer exposure
Longer exposure
Antibodies used:
E-cadherin: Cell signaling, cst3195;
N-cadherin : Cell signaling, cst13116.

## Slide 5
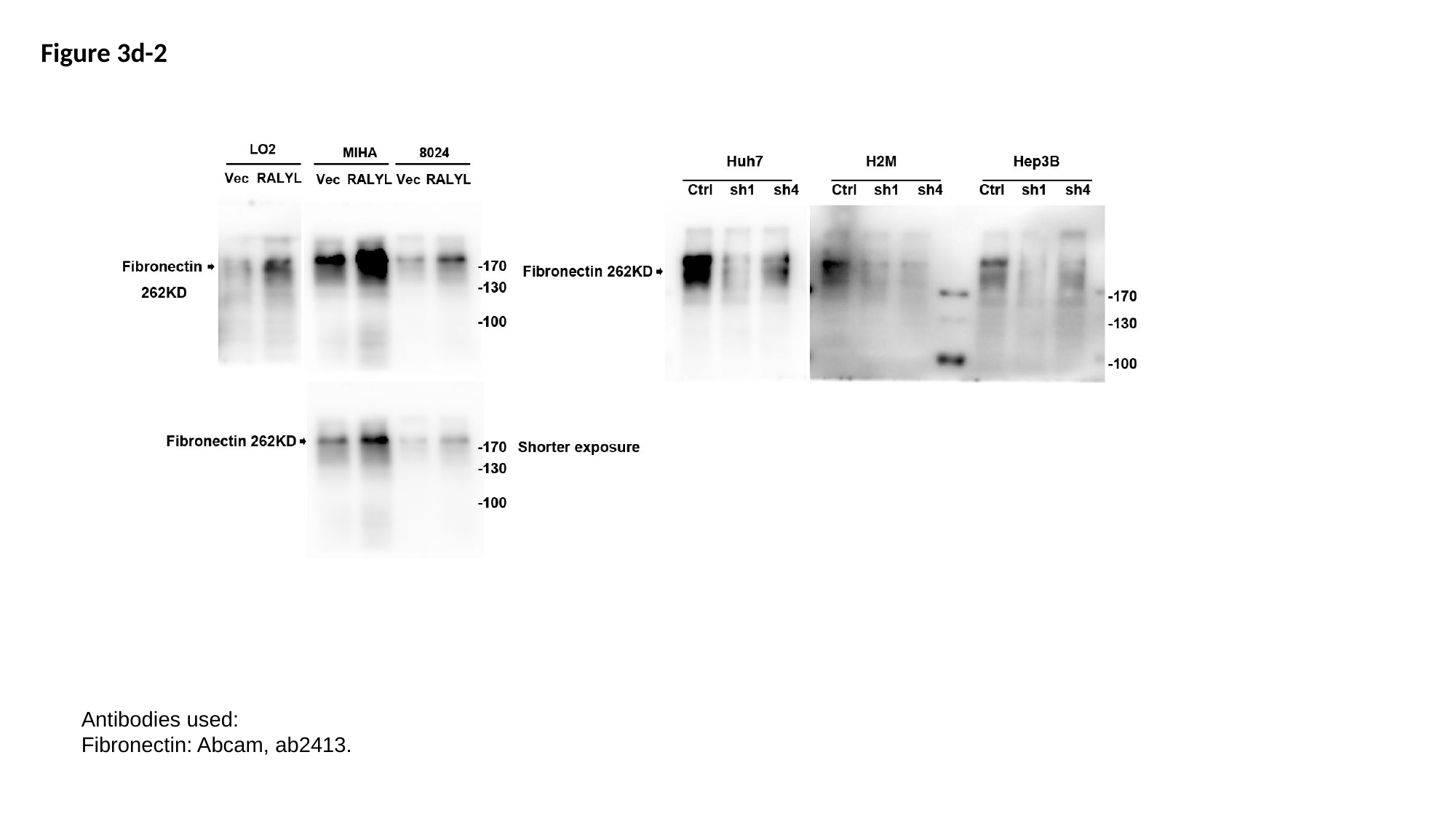

Figure 3d-2
Antibodies used:
Fibronectin: Abcam, ab2413.

## Slide 6
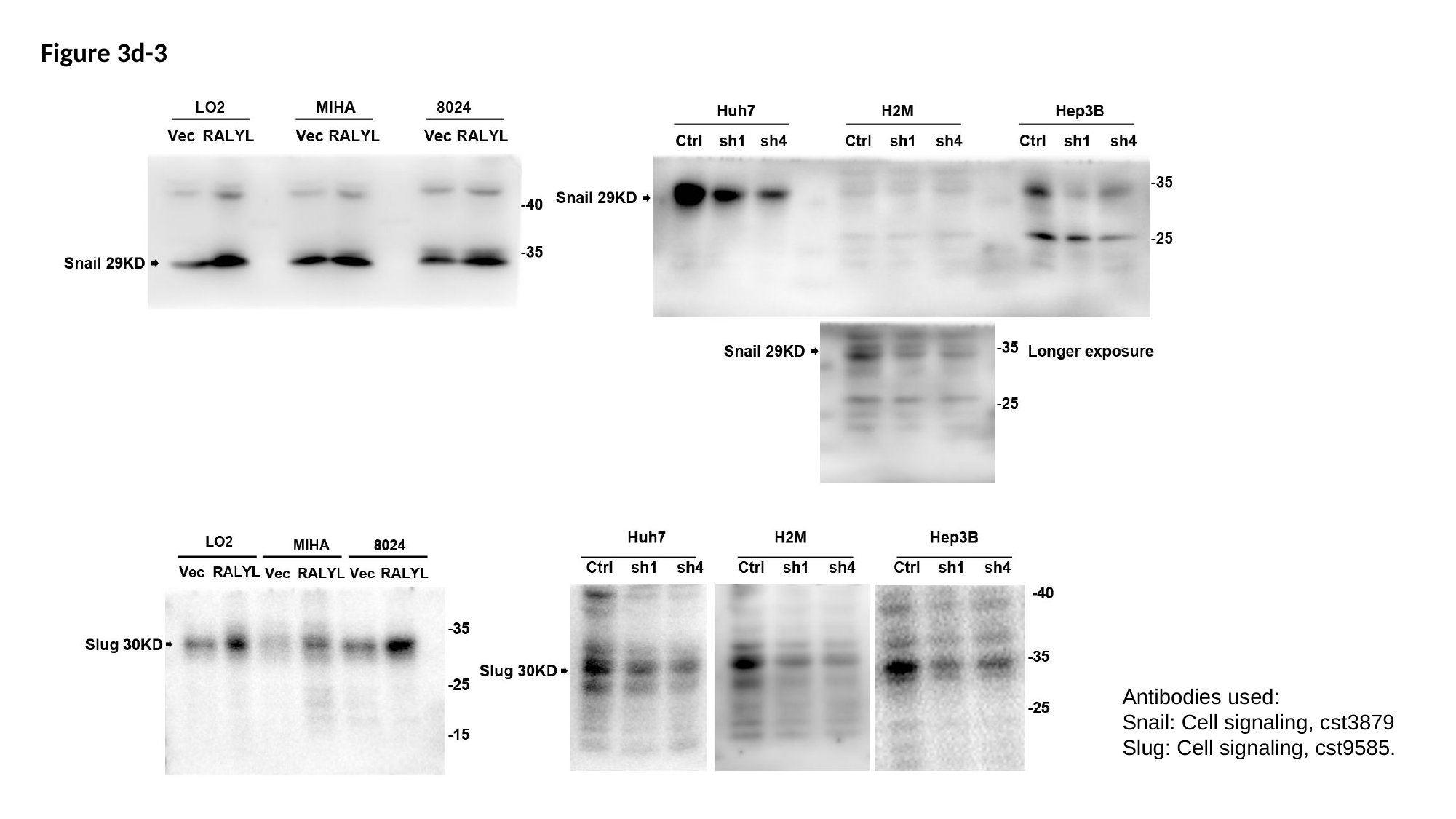

Figure 3d-3
Antibodies used:
Snail: Cell signaling, cst3879
Slug: Cell signaling, cst9585.

## Slide 7
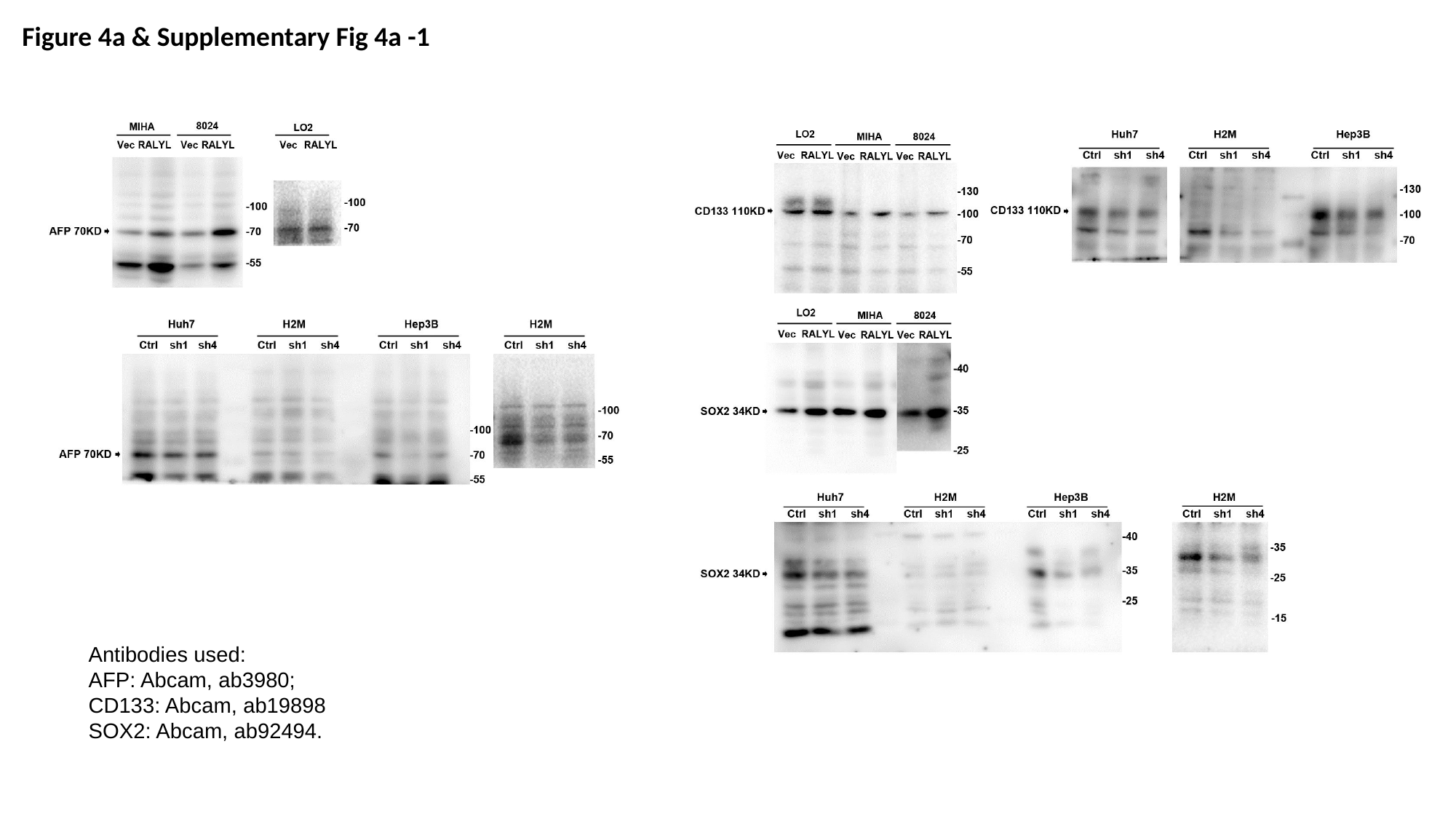

Figure 4a & Supplementary Fig 4a -1
Antibodies used:
AFP: Abcam, ab3980;
CD133: Abcam, ab19898
SOX2: Abcam, ab92494.

## Slide 8
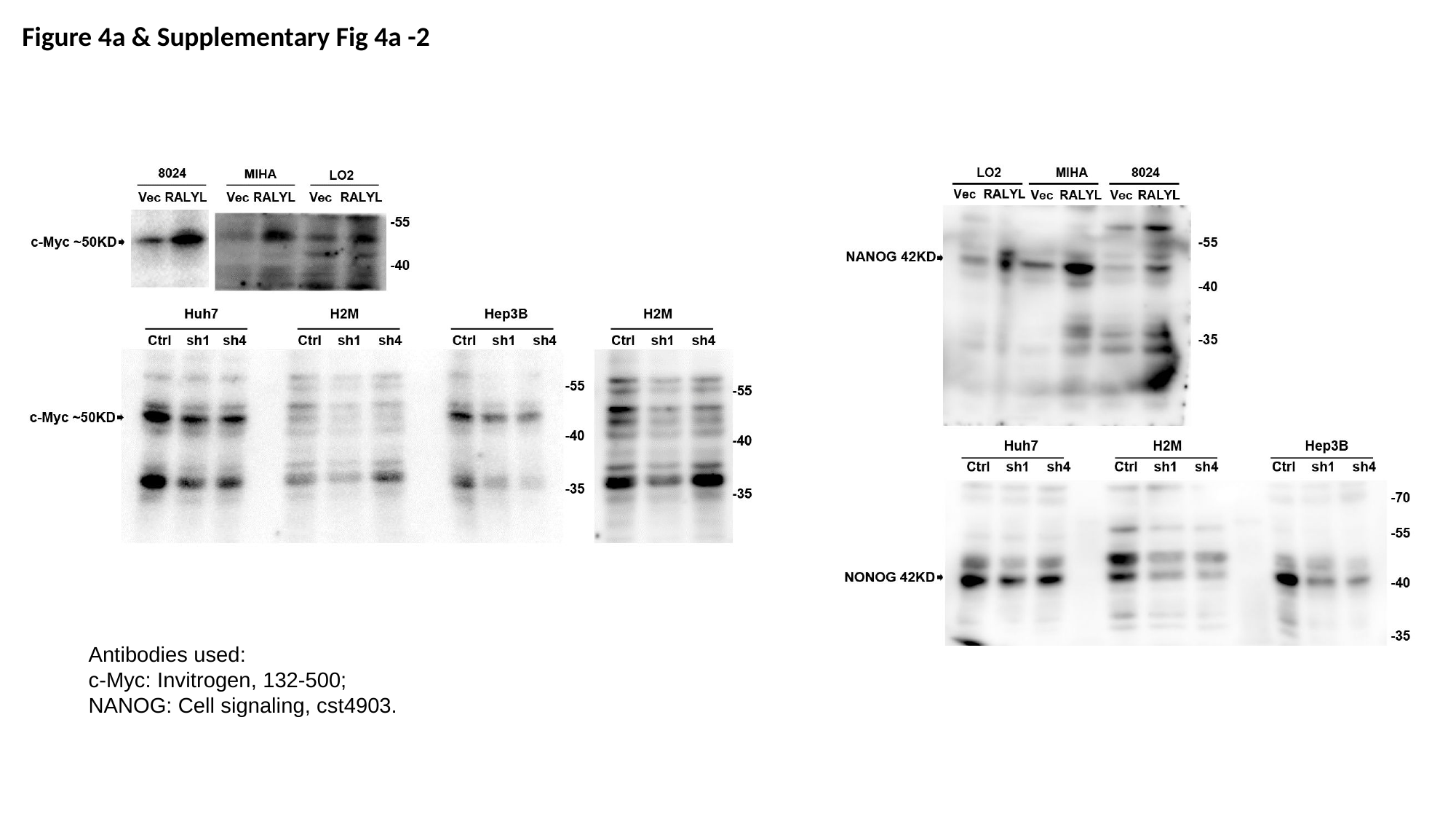

Figure 4a & Supplementary Fig 4a -2
Antibodies used:
c-Myc: Invitrogen, 132-500;
NANOG: Cell signaling, cst4903.

## Slide 9
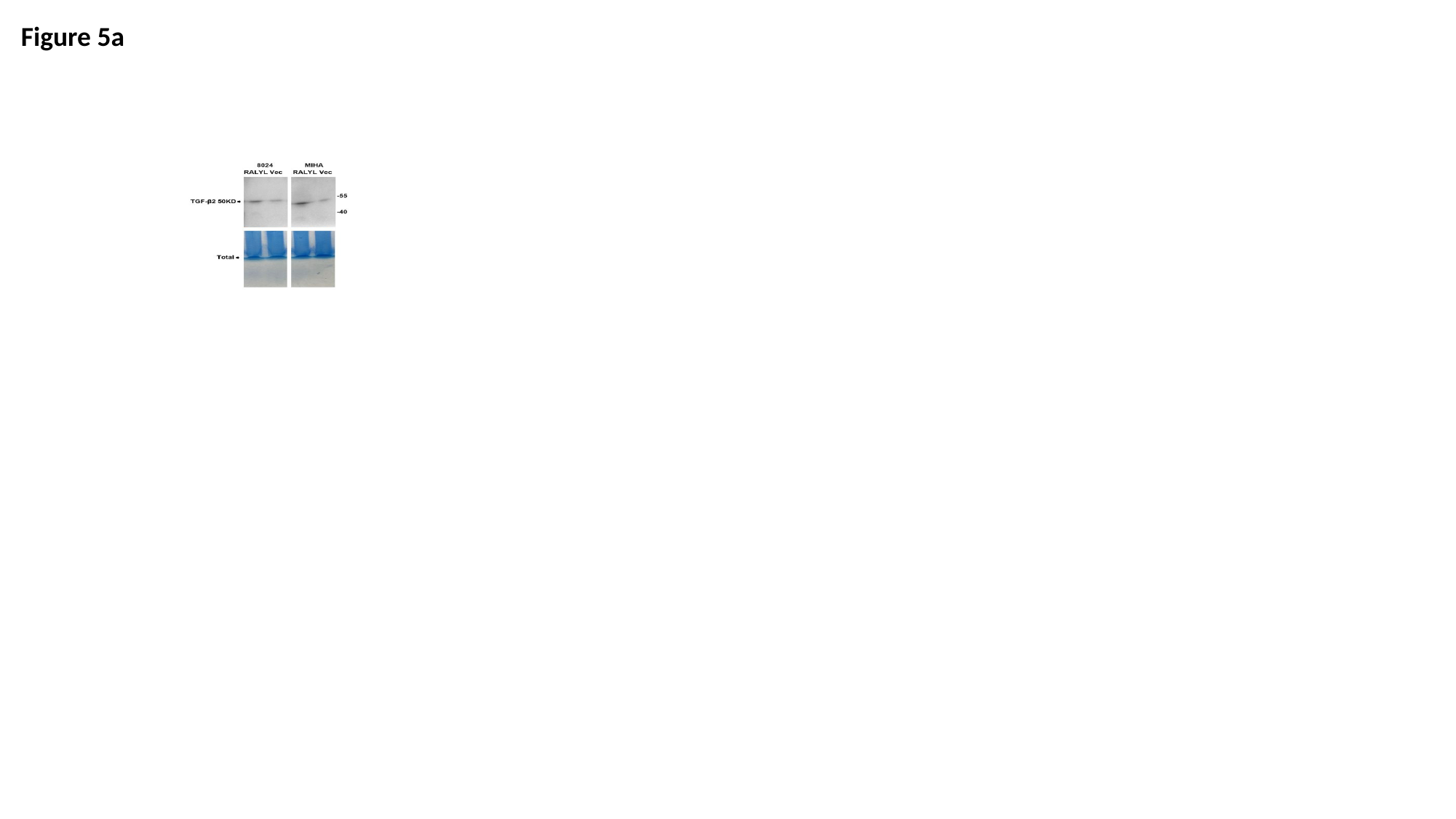

Figure 5a

## Slide 10
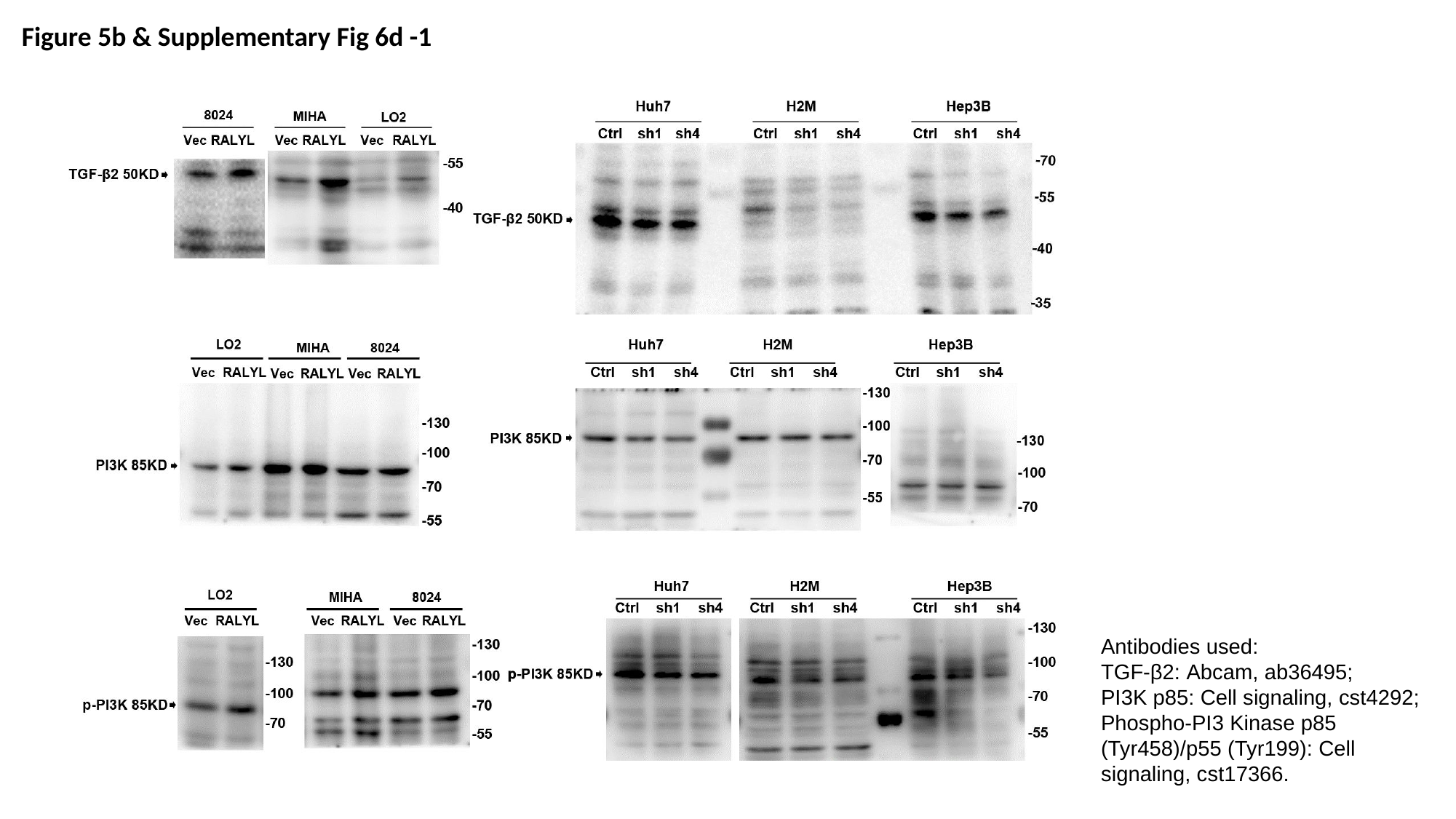

Figure 5b & Supplementary Fig 6d -1
Antibodies used:
TGF-β2: Abcam, ab36495;
PI3K p85: Cell signaling, cst4292;
Phospho-PI3 Kinase p85 (Tyr458)/p55 (Tyr199): Cell signaling, cst17366.

## Slide 11
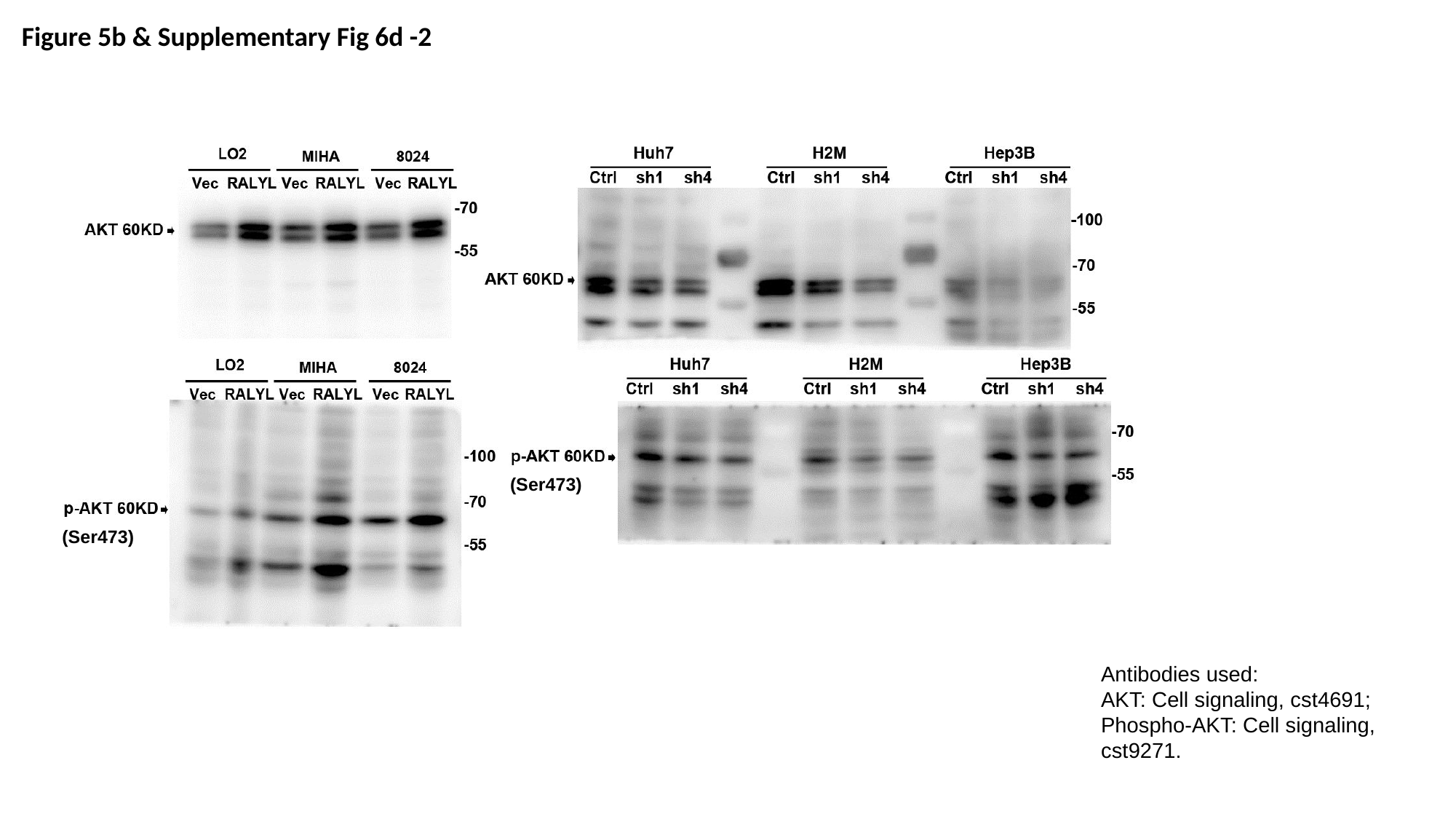

Figure 5b & Supplementary Fig 6d -2
(Ser473)
(Ser473)
Antibodies used:
AKT: Cell signaling, cst4691;
Phospho-AKT: Cell signaling, cst9271.

## Slide 12
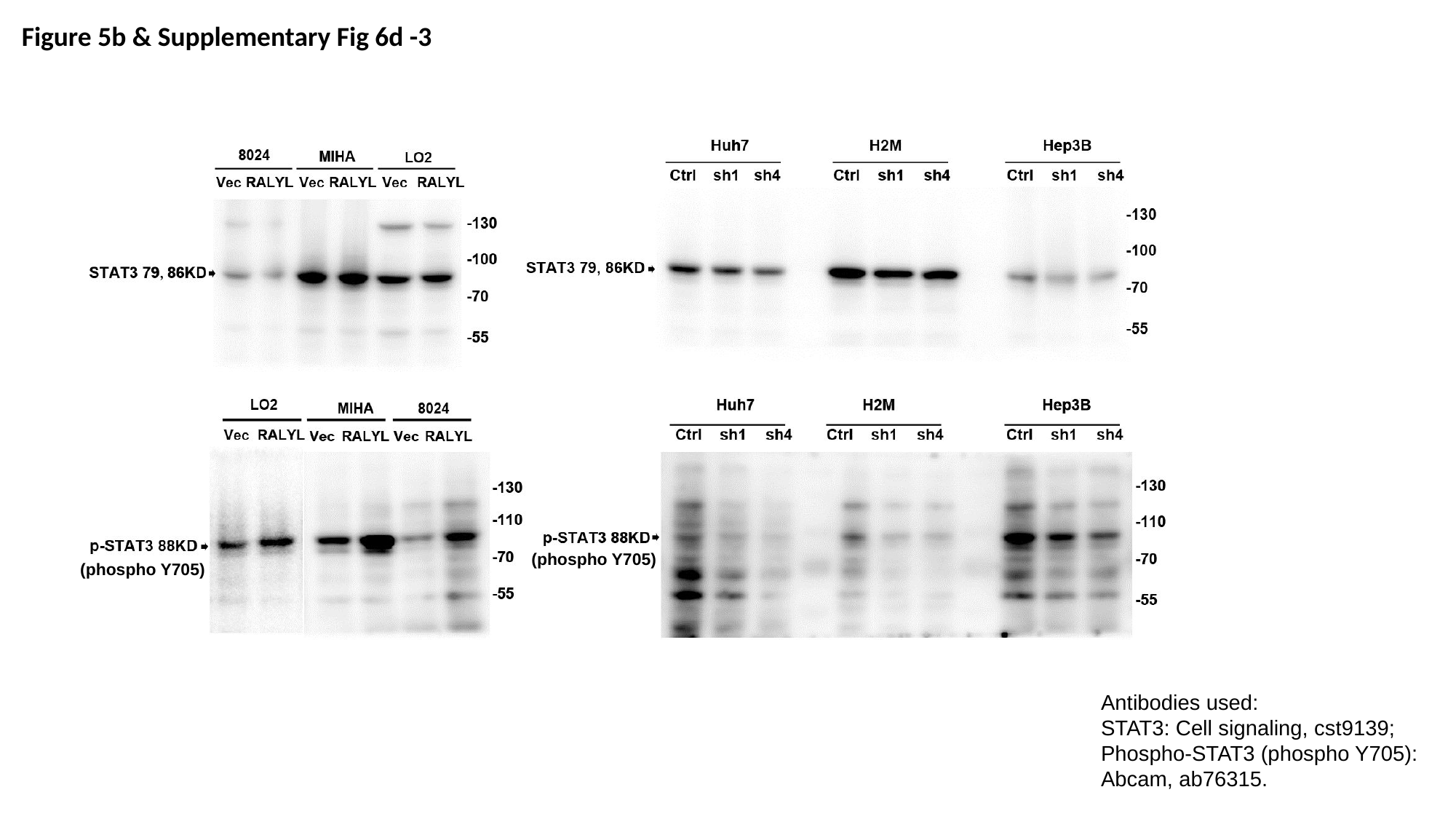

Figure 5b & Supplementary Fig 6d -3
 (phospho Y705)
 (phospho Y705)
Antibodies used:
STAT3: Cell signaling, cst9139;
Phospho-STAT3 (phospho Y705): Abcam, ab76315.

## Slide 13
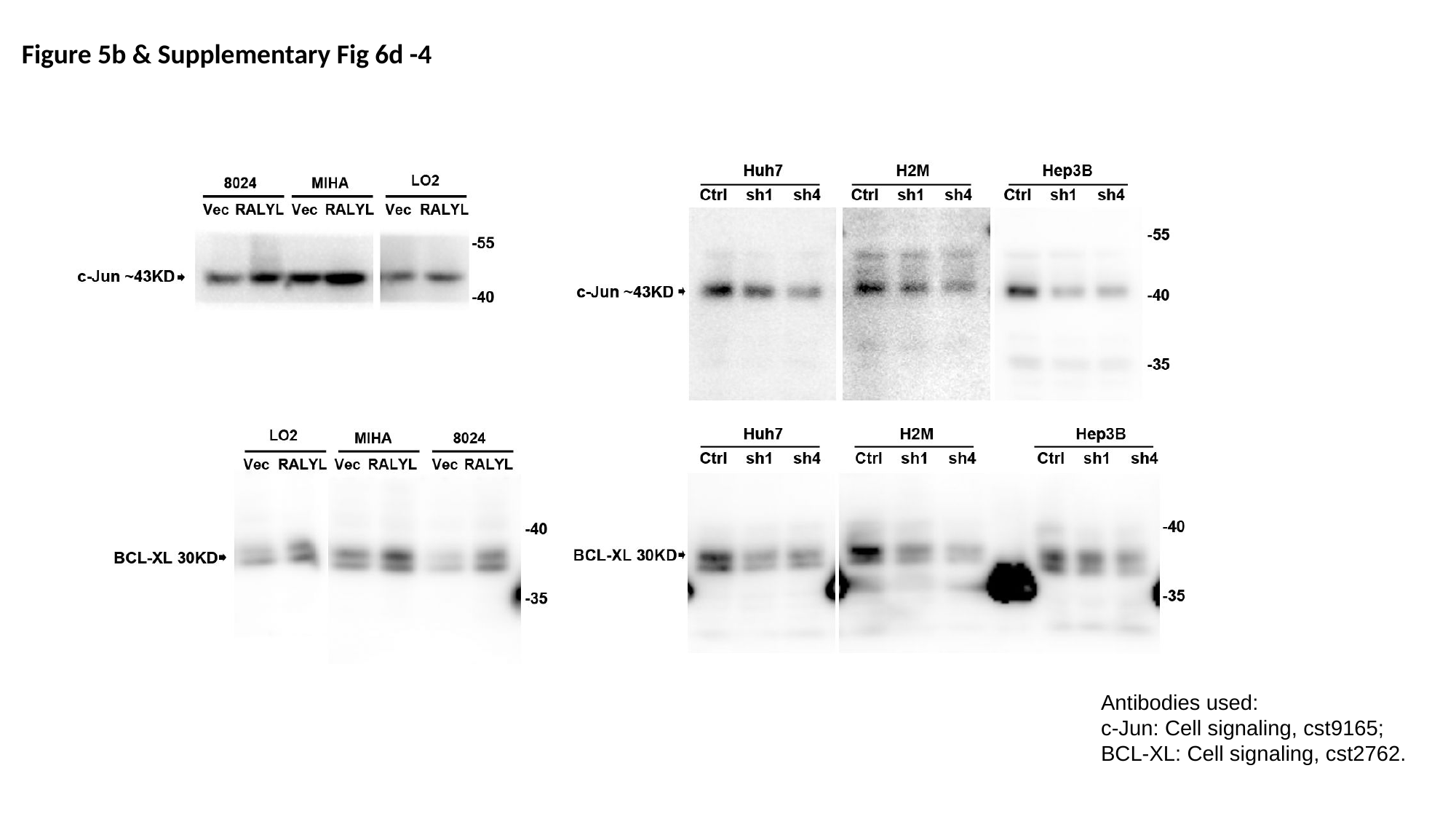

Figure 5b & Supplementary Fig 6d -4
Antibodies used:
c-Jun: Cell signaling, cst9165;
BCL-XL: Cell signaling, cst2762.

## Slide 14
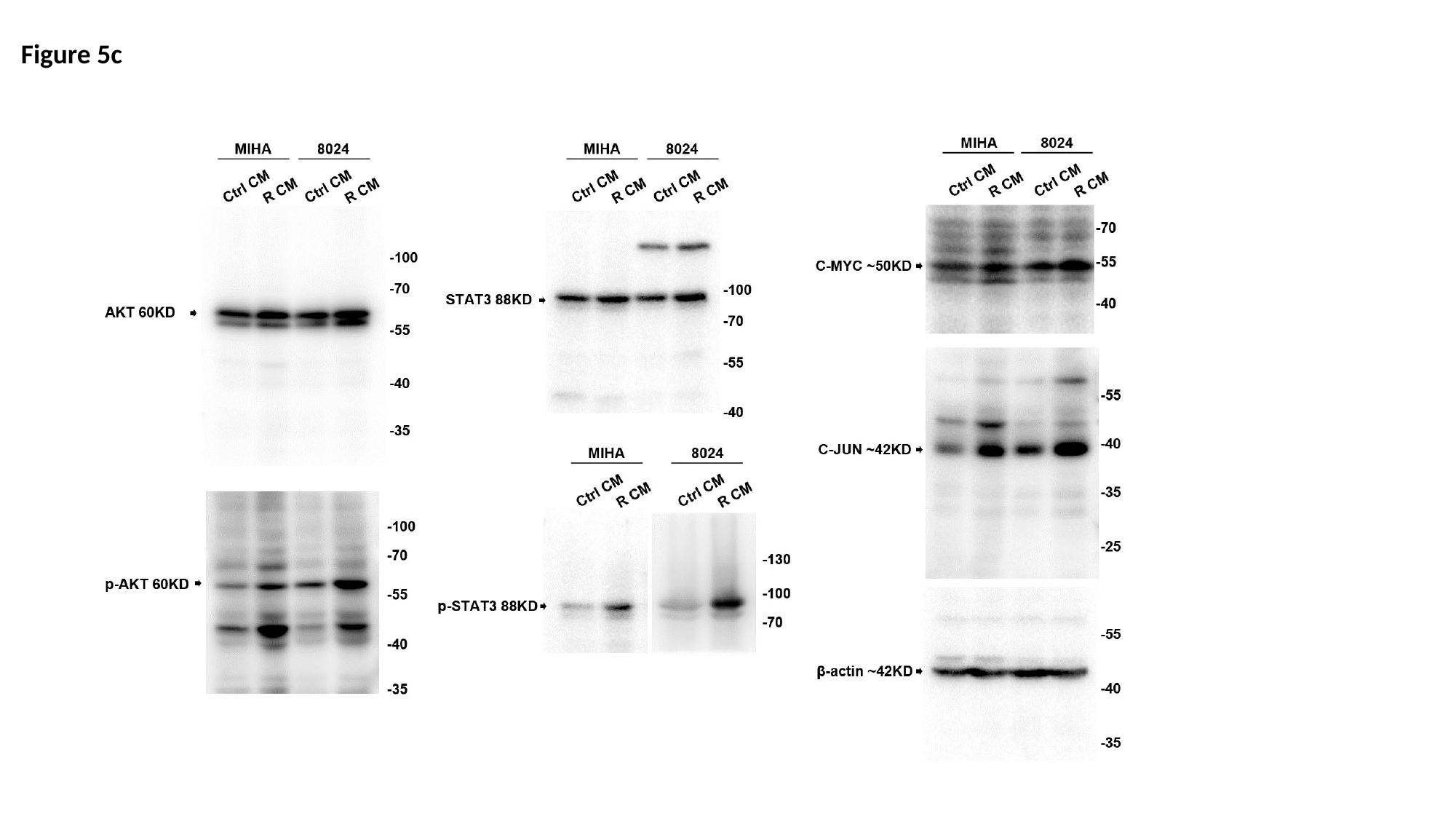

Figure 5c

## Slide 15
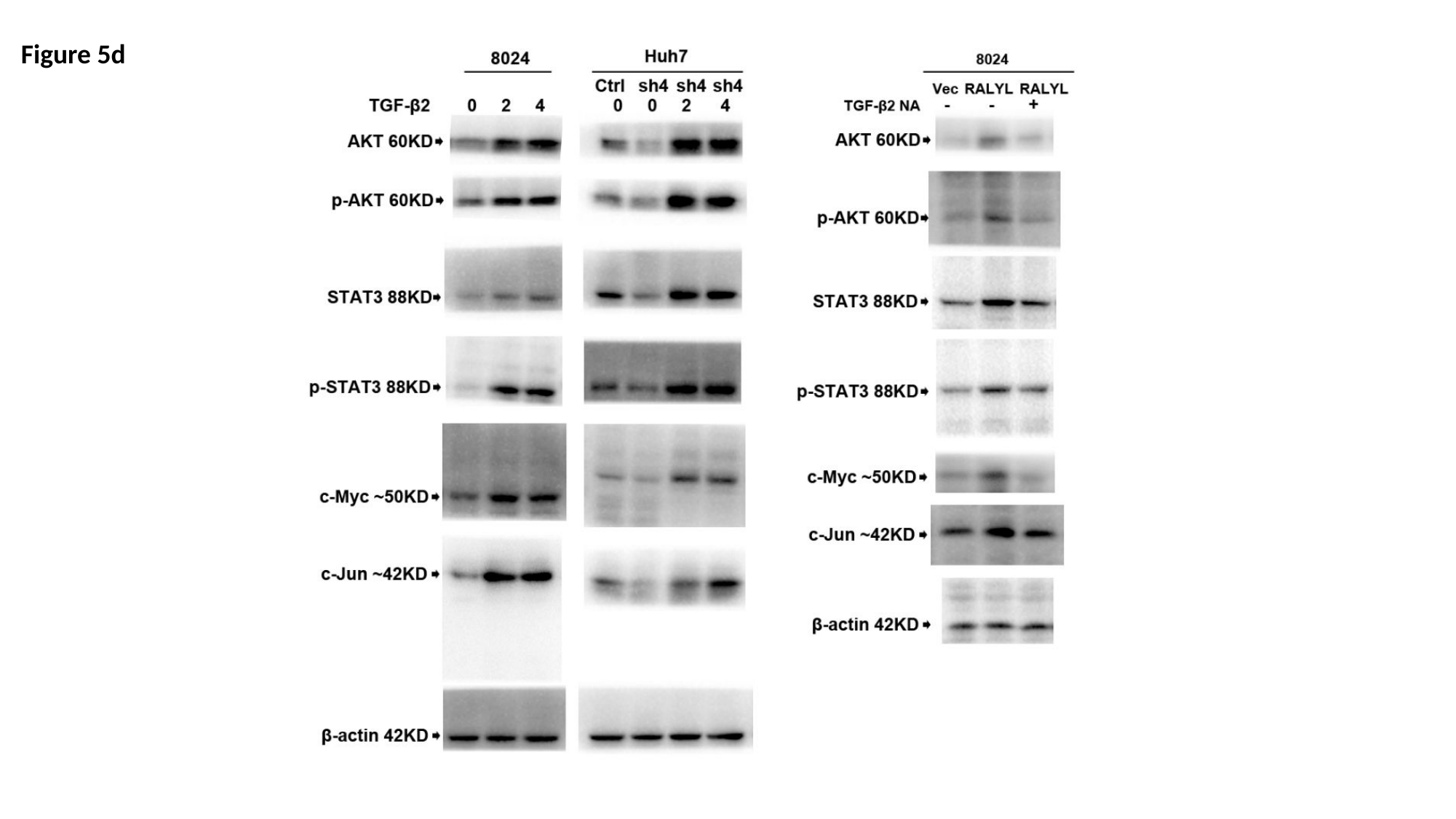

Figure 5d

## Slide 16
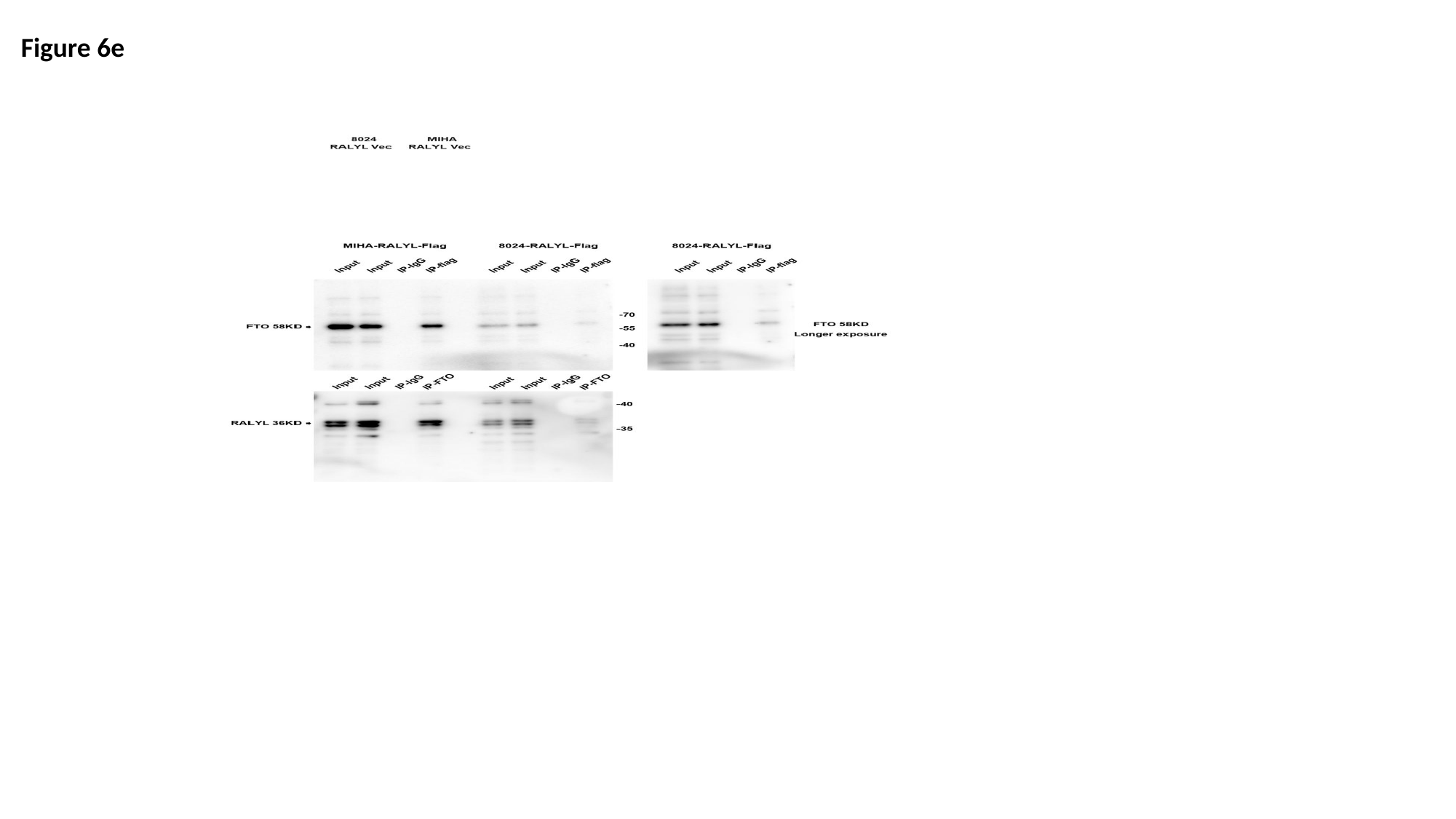

Figure 6e

## Slide 17
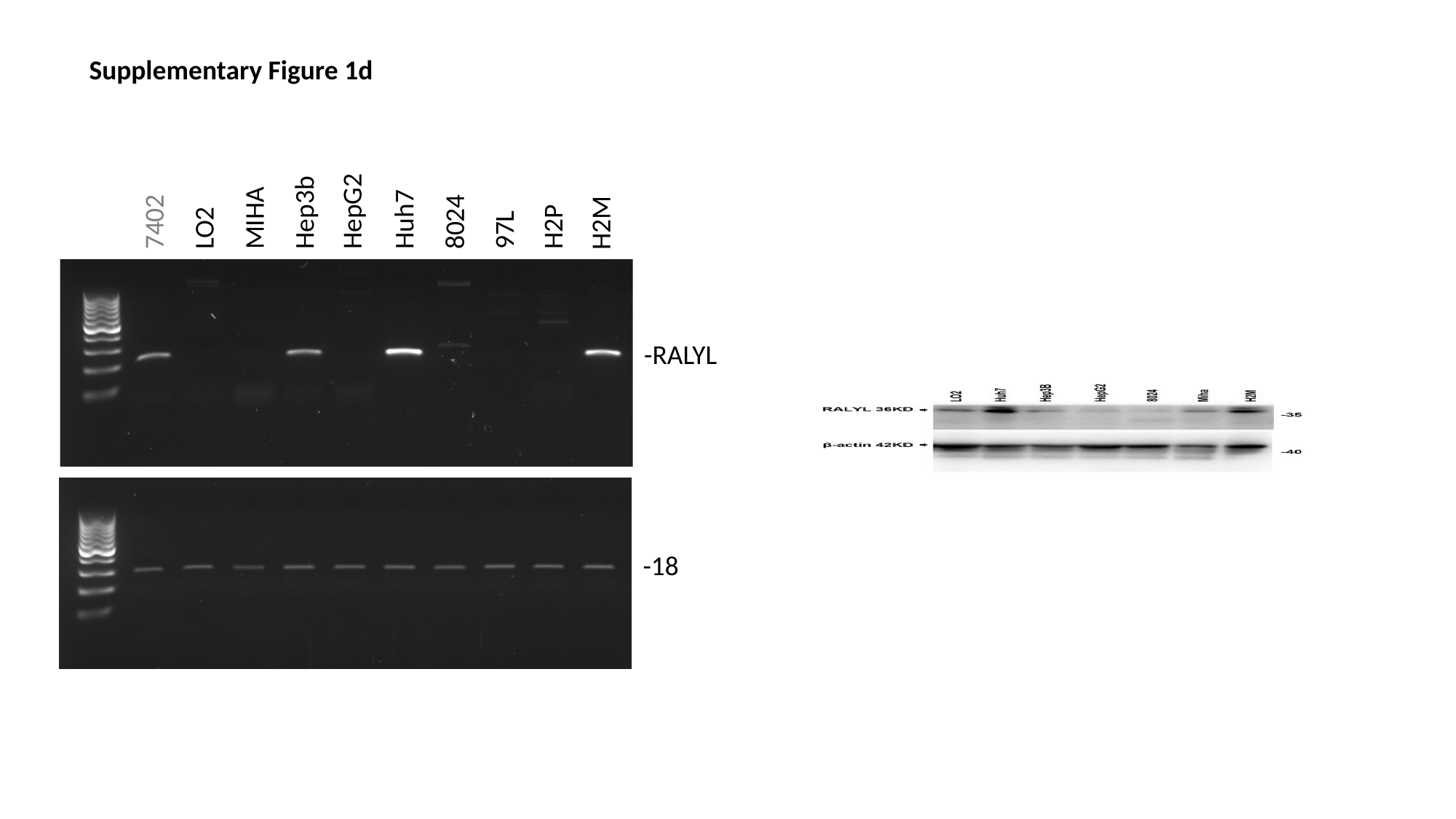

Supplementary Figure 1d
HepG2
Hep3b
H2M
MIHA
Huh7
7402
8024
H2P
LO2
97L
-RALYL
-18
